# ChemR23 Dampens Lung Inflammation and Enhances Anti-viral Immunity in a Mouse Model of Acute Viral Pneumonia

**DOI:** 10.1371/journal.ppat.1002358

**Published:** 2011-11-03

**Authors:** Benjamin Bondue, Olivier Vosters, Patricia de Nadai, Stéphanie Glineur, Olivier De Henau, Souphalone Luangsay, Frédéric Van Gool, David Communi, Paul De Vuyst, Daniel Desmecht, Marc Parmentier

**Affiliations:** 1 Institut de Recherche Interdisciplinaire en Biologie Humaine et Moléculaire (I.R.I.B.H.M.), Faculté de Médecine, Université Libre de Bruxelles, Brussels, Belgium; 2 Service de Pneumologie, Hôpital Erasme, Université Libre de Bruxelles, Brussels, Belgium; 3 Département de Pathologie, Faculté de Médecine Vétérinaire, Université de Liège, Liège, Belgium; 4 Euroscreen SA, Brussels, Belgium; 5 Laboratoire de Physiologie Animale, Institut de Biologie et de Médecine Moléculaires, Université Libre de Bruxelles, Gosselies, Belgium; University of Pennsylvania School of Medicine, United States of America

## Abstract

Viral diseases of the respiratory tract, which include influenza pandemic, children acute bronchiolitis, and viral pneumonia of the elderly, represent major health problems. Plasmacytoid dendritic cells play an important role in anti-viral immunity, and these cells were recently shown to express ChemR23, the receptor for the chemoattractant protein chemerin, which is expressed by epithelial cells in the lung. Our aim was to determine the role played by the chemerin/ChemR23 system in the physiopathology of viral pneumonia, using the pneumonia virus of mice (PVM) as a model. Wild-type and ChemR23 knock-out mice were infected by PVM and followed for functional and inflammatory parameters. ChemR23^−/−^ mice displayed higher mortality/morbidity, alteration of lung function, delayed viral clearance and increased neutrophilic infiltration. We demonstrated in these mice a lower recruitment of plasmacytoid dendritic cells and a reduction in type I interferon production. The role of plasmacytoid dendritic cells was further addressed by performing depletion and adoptive transfer experiments as well as by the generation of chimeric mice, demonstrating two opposite effects of the chemerin/ChemR23 system. First, the ChemR23-dependent recruitment of plasmacytoid dendritic cells contributes to adaptive immune responses and viral clearance, but also enhances the inflammatory response. Second, increased morbidity/mortality in ChemR23^−/−^ mice is not due to defective plasmacytoid dendritic cells recruitment, but rather to the loss of an anti-inflammatory pathway involving ChemR23 expressed by non-leukocytic cells. The chemerin/ChemR23 system plays important roles in the physiopathology of viral pneumonia, and might therefore be considered as a therapeutic target for anti-viral and anti-inflammatory therapies.

## Author Summary

Infections of the lower respiratory tract by single-stranded RNA viruses represent a major health problem worldwide. Animal models indicate that the severity of infections caused by these viruses is due essentially to an excessive primary immune response of the host, rather than the direct cytopathogenicity of the viruses. Plasmacytoid dendritic cells have been reported to play an important role in anti-viral immunity, but the factors responsible for the recruitment of these cells to the infected lung were unknown. This study depicts the roles of the G protein-coupled receptor ChemR23 in the recruitment of plasmacytoid dendritic cells and anti-viral immunity in a mouse model of acute viral pneumonia. The data also highlight the role of ChemR23 in dampening the lung inflammatory response. This latter effect is independent of pDC recruitment but involves non-leukocytic cells. This observation is of particular interest considering the established role of airway endothelial and epithelial cells in the immune responses following bacterial, viral and fungal infections. Our results suggest therefore that the chemerin/ChemR23 system might be considered as a target for anti-viral and anti-inflammatory therapies.

## Introduction

A number of single-stranded RNA viruses, including influenza and respiratory syncytial virus (RSV) cause infections of the lower respiratory tract and pneumonias, representing thereby a major health problem worldwide. These viruses constitute a major cause of hospitalization for acute bronchiolitis in infants and young children (especially RSV), exacerbation of chronic obstructive pulmonary disease (COPD), and viral pneumonia in the elderly [1]–[4]. Animal models indicate that the severity of infections caused by RSV or its mouse counterpart, the pneumonia virus of mice (PVM), is due essentially to an excessive primary immune response of the host, rather than the direct cytopathogenicity of the viruses [5]–[8]. In line with these observations, administration of anti-viral drugs was shown to have minimal impact on the clinical outcome. Following an early and non-specific inflammatory response characterized by the recruitment of neutrophils and cytotoxic natural killer cells [9]–[10], a delayed adaptive immune response leads, 7 to 10 days after infection, to an important influx of CD8^+^ cytotoxic T cells in the lung, and the production of RSV- or PVM-specific antibodies by plasma cells [5], [8], [10]. Dendritic cells (DCs) play a central role in the initiation of this adaptive immune response. DCs capture viral antigens in the lung, undergo a maturation process, and migrate to lymph nodes where they activate naive CD4^+^ and CD8^+^ T cells [11]–[12]. In the lung, two major subsets of dendritic cells have been described: CD11c^+^ CD11b^+^ MHC II^high^ F4-80^−^ conventional, or myeloid, DCs (cDC or mDC) and CD11c^low^ CD11b^−^ Gr1^+^ mPDCA-1^+^ plasmacytoid DCs (pDC) [13]–[16]. In contrast with myeloid DCs, plasmacytoid DCs display a relatively weak antigen-presenting capability but exert essential immuno-modulatory functions in asthma and viral diseases [17]. PDCs are major producers of type I interferons (IFNs), including IFN-α and IFN-β, in response to the sensing of viral components through Toll-like receptors (TLRs). Type I IFNs play a crucial role in innate anti-viral immunity, but also in the initiation of adaptive immune responses [18]. Nevertheless, the role of pDCs in the physiopathology of viral pneumonia is still unclear, as discrepancies exist among published data. Indeed, some studies performed with influenza virus or human RSV suggest a limited role for pDCs [16], [19], [20] whereas depletion and adoptive transfer experiments have demonstrated the ability of pDCs to inhibit viral replication and enhance viral clearance in RSV-infected mice. They also decrease airway hyperreactivity, lung inflammation and mucus production, properties attributed to inherent anti-inflammatory properties of pDCs [21], [22]. Such anti-inflammatory activity of pDCs is also described in asthma, in which pDCs mediate tolerance through induction of regulatory T lymphocytes and suppression of effector T cell generation [14], [23]–[25].

ChemR23 is a G protein-coupled receptor, expressed by immature DCs, macrophages and NK cells [26]–[28]. Its natural ligand, chemerin, was purified from human inflammatory fluids [29]. Chemerin, acting through ChemR23, has chemoattractant properties at low and subnanomolar concentrations, particularly for immature plasmacytoid DCs (pDCs) that express ChemR23 at high levels [28], [30], [31]. Chemerin is synthesized as an inactive precursor, prochemerin, which is present at high concentration in plasma. Prochemerin can be rapidly converted into a full ChemR23 agonist by neutrophil-derived serine proteases (elastase and cathepsin G) and serine proteases of the coagulation and fibrinolytic cascades, released as a result of tissue injury, inflammation or infection [32], [33].

Provided the expression of prochemerin in lung [28], [29], its processing by neutrophil proteases, the preferential expression of ChemR23 by pDCs, and the role of pDCs and neutrophils in the physiopathology of viral infections of the lung, we investigated the potential involvement of the chemerin-ChemR23 system in pDC recruitment and its functional consequences during viral pneumonia. We used wild-type (WT) and ChemR23 knock-out (KO) mice in the PVM-induced acute pneumonia model and demonstrated a crucial role of ChemR23 in the recruitment of pDCs to the lung, the control of viral replication and clearance, as well as in the dampening of excessive inflammatory responses. The enhanced inflammatory status and higher mortality rate of infected ChemR23-deficient mice is however not due to the impairment of pDC recruitment, but rather to the loss of an anti-inflammatory role of chemerin through non-leukocytic cells.

## Results

### Enhanced mortality, morbidity, and respiratory dysfunction in infected ChemR23^−/−^ mice

**Figure 1:**
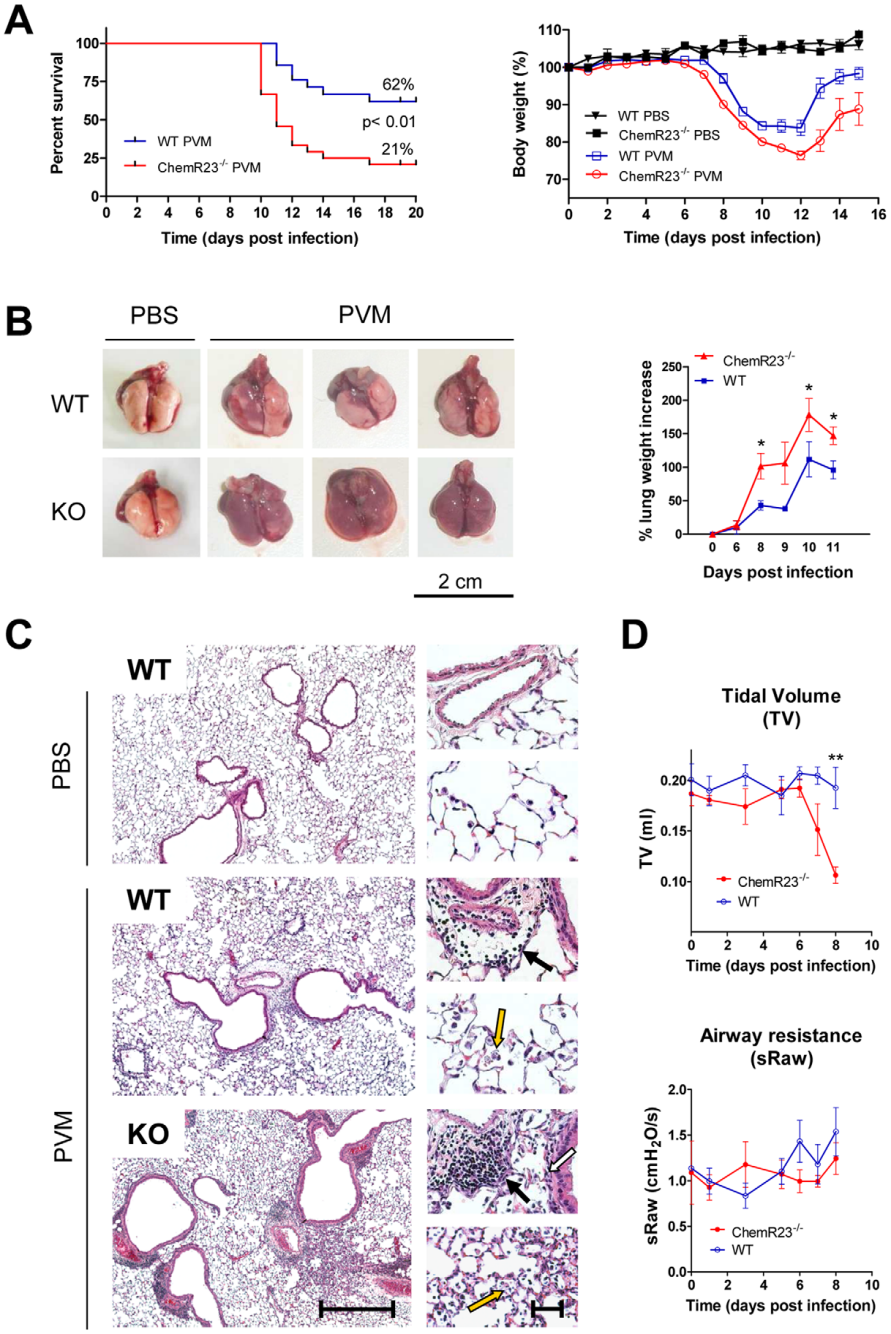
Higher mortality rate and more severe pathological features in ChemR23^−/−^ mice infected by PVM. (**A**) Following intranasal inoculation of PVM (1000 PFUs) or control medium, ChemR23^−/−^ and wild-type (WT) C57BL/6 mice were monitored daily for survival (left panel) and weight loss (right panel). Weight curves (mean ± SEM) are relative to initial body weight. Differences were significant (p<0.05) between infected wild-type (blue curve) and ChemR23^−/−^ (red curve) mice from day 7 to day 16. The displayed data result from the pooling of four independent experiments (n = 40 for infected groups and n = 10 for uninfected groups). (**B**) Macroscopic aspect of lungs collected from WT and ChemR23^−/−^ (KO) mice 9 days after PVM infection or PBS instillation. The weight of collected lungs was assessed at various time-points post-infection (right panel) and expressed as percentage over lung weight of uninfected control mice. The data shown are the mean ± SEM for groups of seven animals. (**C**) Representative sections of lungs stained with hematoxylin and eosin. Scale bars: 500 µm for left panels, 50 µm for right panels. PVM-infected lungs display perivascular (black arrows), peribronchiolar (white arrow), and alveolar inflammation (yellow arrows). (**D**) Respiratory dysfunction was measured using a double chamber plethysmograph. Before and at selected time points after intranasal inoculation of PVM, tidal volume (TV) and specific airway resistance (sRaw) were determined in wild-type and ChemR23^−/−^ mice. The data shown are the mean ± SEM for groups of four animals. All displayed data are representative of at least three independent experiments. *, p<0.05; **, p<0.01.

After intranasal inoculation of PVM (1000 PFU) or control medium (PBS), ChemR23^−/−^ and wild-type C57BL/6 mice were monitored daily for survival and weight loss. A significantly lower survival rate was observed in ChemR23^−/−^ mice as compared to WT mice (21% vs 62% respectively; p<0.01) (Figure 1A). ChemR23^−/−^ mice began to lose weight one day ahead of WT mice (i.e. at days 7 and 8 respectively, Figure 1A). Thereafter, the weight curves were grossly parallel up to day 10, when WT mice stabilized their weight before recovering, whereas ChemR23^−/−^ mice continued to lose weight till day 12 included. Furthermore, at days 8 and 9 post-infection, infected ChemR23^−/−^ mice presented a more severe pattern of illness signs, characterized by motor slowing, hunching, fur ruffling, and crackles. It should also be noted that the difference in weight loss remained significant at late time points (days 12 to 16), when the more affected animals had died, particularly in the infected ChemR23^−/−^ group.

The differences in mortality rate and weight loss between infected ChemR23^−/−^ and WT mice correlated with the pathological findings made on lungs collected at day 9 post-infection (Figure 1B and 1C). Macroscopic examination of infected lungs revealed numerous erythematous inflammatory foci, which were larger and more numerous in Chem23^−/−^ mice than in controls (Figure 1B). As expected, such foci were never seen in uninfected mice. At selected time points after infection, the lung weight was recorded, as a reflect of inflammation and edema. ChemR23^−/−^ mice presented a higher lung weight increase (44% over that of WT mice on average between day 6 and day 11) (Figure 1B). Microscopic examination of the lungs clearly showed stronger interstitial, peribronchiolar, and perivascular infiltrates rich in neutrophils, lymphocytes and macrophages in infected ChemR23^−/−^ mice (Figure 1C).

Next, we investigated the respiratory function of the animals, using a whole body double chamber plethysmograph (Figure S1). No significant changes were observed up to day 6 post-infection. At days 7 and 8 post-infection, while WT mice exhibited limited changes of the various parameters, ChemR23^−/−^ mice displayed a severe restrictive syndrome likely resulting from the strong lung edema and leukocyte infiltrate. This was characterized by a sharp decrease (up to 50%) in tidal volume (p<0.01), a 2.3-fold increase in end expiratory pause (p<0.05), a 3.4-fold increase in enhanced pause (Penh) (p<0.001), and a 65% reduction in expiratory balance (p<0.05) (Figure 1D and Figure S1). No difference in airway resistance (sRaw) was observed between ChemR23^−/−^ and WT mice (Figure 1D). Plethysmographic analysis could not be performed after day 8 post-infection, as a result of bioethical limitations caused by the viral disease.

### Chemerin increases in airways during viral infection

**Figure 2:**
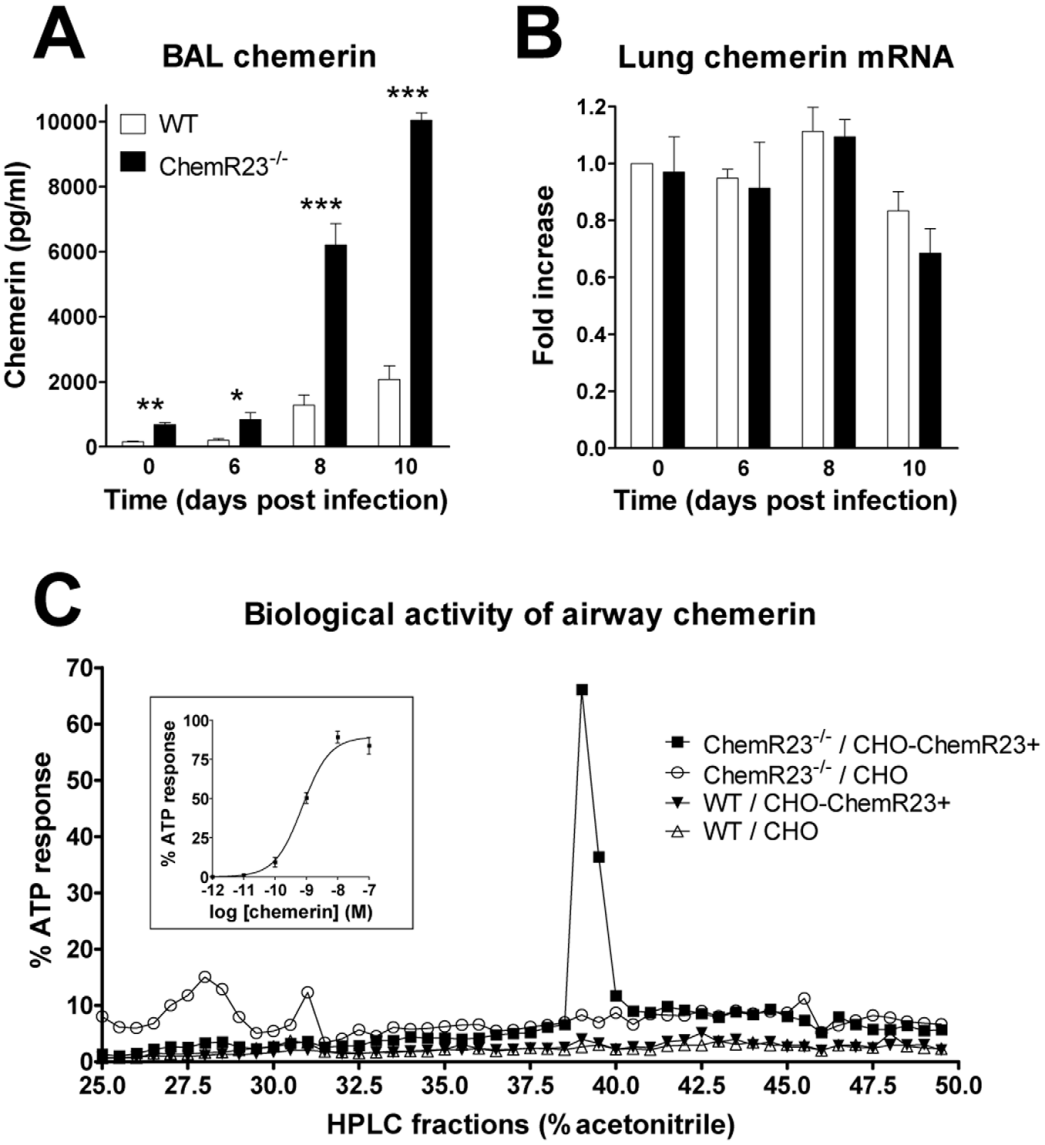
Increased chemerin levels in broncho-alveolar lavage fluid during PVM infection. (**A**) At selected time points after viral inoculation, chemerin levels were determined by ELISA in BAL fluids of ChemR23^−/−^ and wild-type C57BL/6 mice. (**B**) Chemerin transcript levels were determined by quantitative RT-PCR. The data were normalized using two housekeeping genes (YWHAZ and CANX) as references, and reported to the chemerin transcript level in uninfected control mice. The displayed data are the mean ± SEM for groups of minimum seven animals. (**C**) The biological activity of chemerin was measured in BAL fluids obtained at day 9 post-infection from wild-type (WT) and ChemR23^−/−^ mice after a reverse phase HPLC fractionation (C18 column) using an aequorin-based intracellular Ca^2+^ mobilization assay. The data shown are the activity of fractions (25 to 50% acetonitrile) normalized to the response to ATP upon testing on CHO-K1 cells expressing mouse ChemR23 (CHO-ChemR23+) or not (CHO). The functional response of mouse ChemR23-expressing CHO-K1 cells to recombinant mouse chemerin is shown as inset. Data are representative of three independent experiments. *, p<0.05; **, p<0.01; ***, p<0.001.

Chemerin, the agonist of ChemR23, was assayed by ELISA in the broncho-alveolar lavage at several time points after infection. Chemerin levels increased from day 8 to day 10 post-infection in both ChemR23^−/−^ and WT mice (Figure 2A). However, they were higher (4.5-fold on average) in ChemR23^−/−^ mice at each time point with the highest levels at day 10 (10.0±0.2 versus 2.1±0.4 ng/ml for WT, p<0.001). In order to investigate whether elevated chemerin levels were the consequence of increased local expression, chemerin transcripts were assessed by qRT-PCR (Figure 2B). The data were normalized using two housekeeping genes (YWHAZ and CANX) as references, and reported to the chemerin transcript level in uninfected control mice. No significant differences were observed according to infection status or genotype. As the ELISA assays both active and inactive forms of chemerin (including prochemerin), we tested chemerin bioactivity in BAL fluids. Fractions resulting from a reverse phase HPLC were tested in an aequorin-based intracellular Ca^2+^ mobilization assay. A biological activity was recorded for fractions obtained from infected ChemR23^−/−^ mice using CHO-K1 cells expressing mouse ChemR23 (Figure 2C). Bioactivity was also retrieved from infected WT mice, following sample concentration, while no activity was observed in fractions from naive mice (data not shown).

### Increased viral load and delayed viral clearance in ChemR23^−/−^ mice

**Figure 3:**
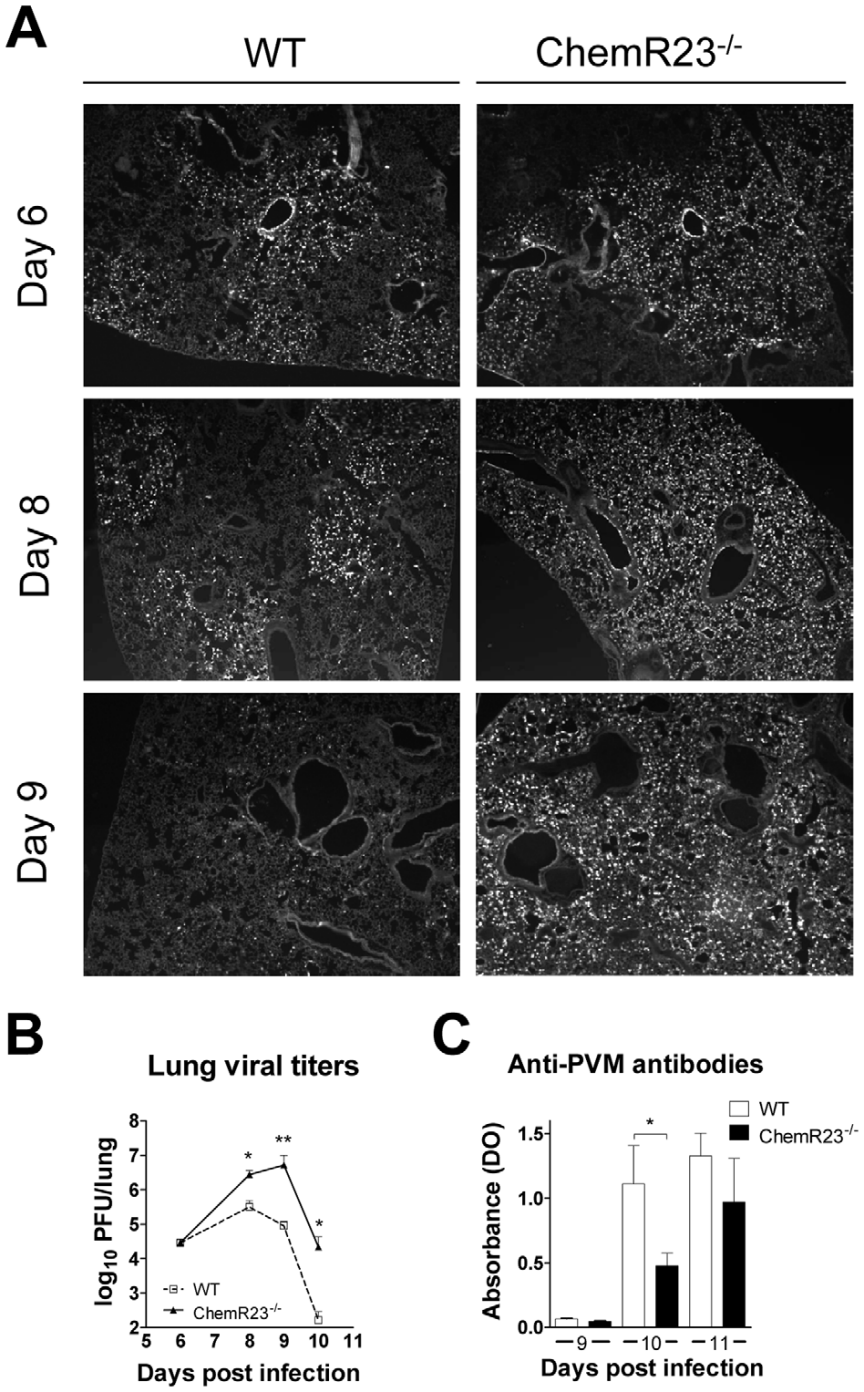
Increased viral load and delayed viral clearance in ChemR23^−/−^ mice. (**A**) Immunofluorescent staining of viral antigens in lung sections at 6, 8, and 9 days post-infection of wild-type (WT) and ChemR23^−/−^ mice. Original magnification: ×50. (**B**) Viral titers were determined in lung homogenates for WT (open squares) and ChemR23^−/−^ (filled triangles) mice at selected time points post-infection, and expressed in log_10_ plaque-forming units (PFU) per lung. (**C**) At days 9, 10, and 11 post-infection, the serum of infected mice was assessed for the presence of anti-PVM antibodies by ELISA. The displayed data are the mean ± SEM for groups of seven animals and are representative of at least two independent experiments. *, p<0.05; **, p<0.01.

To assess the role of ChemR23 in viral replication and clearance, immunofluorescence staining of lung sections with an anti-PVM antibody was performed at days 6, 8, and 9 post-infection. This allowed the observation of PVM-infected cells, which include bronchiolar epithelial cells, type I and type II pneumocytes, alveolar macrophages and eosinophils [34]–[36 and unpublished observations] (Figure 3A). No noticeable difference was observed at day 6 between WT and ChemR23^−/−^ mice. Thereafter, PVM staining remained stable in WT mice at day 8, and decreased by day 9, whereas it increased in ChemR23^−/−^ mice up to day 9. As a result, ChemR23^−/−^ sections showed a stronger and more diffuse staining than WT mice at day 8, and the difference increased further by day 9. The enhanced viral replication and reduced viral clearance in ChemR23^−/−^ mice were confirmed by the determination of the lung viral titers by plaque assay (Figure 3B). The peak of viral titer was reached later in ChemR23^−/−^ than in WT mice (respectively at day 8 and 10 post-infection), and the titers were up to 100-fold higher in ChemR23^−/−^ than in WT mice (2.7±0.9×10^6^ versus 0.31±0.16×10^6^ PFU/lung at day 8, p<0.05; 5.2±4.5×10^6^ versus 9.1±2.9×10^4^ PFU/lung at day 9, p<0.01; 2.2±2.1×10^4^ versus 170±100 PFU/lung at day 10, p<0.05 respectively). As for immunostaining, no difference in viral titers was apparent at day 6 (2.9±0.8×10^4^ for ChemR23^−/−^ mice and 2.9±0.6×10^4^ PFU/lung for WT mice, p>0.05). The delayed viral clearance in ChemR23^−/−^ mice was associated with a mild decrease in anti-PVM antibodies in sera of ChemR23^−/−^ mice at day 10 post-infection, whereas this difference was abrogated at day 11 (Figure 3C).

### Impaired type I IFN and IL-12p40 synthesis in infected ChemR23^−/−^ mice

**Figure 4:**
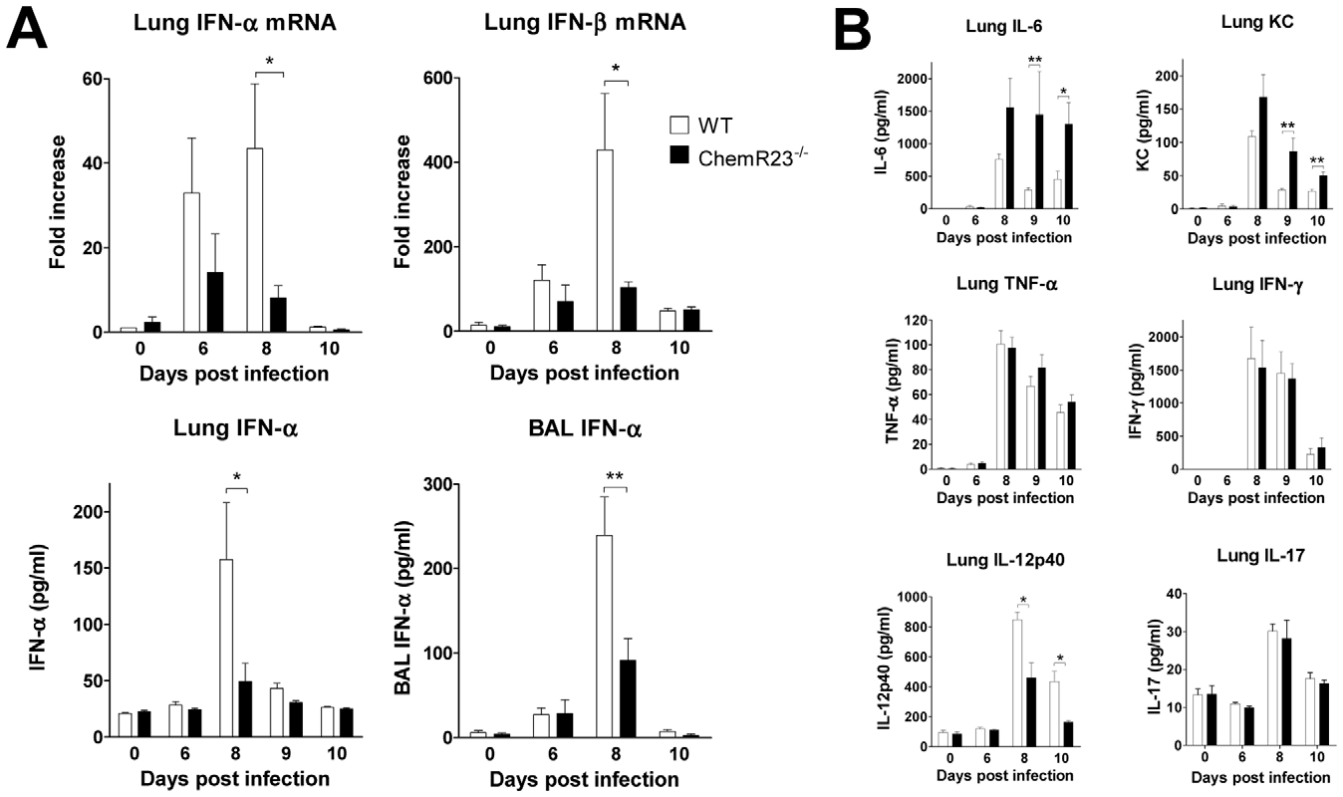
Reduced levels of type I IFNs and IL-12p40 in ChemR23^−/−^ infected mice. At selected time points after infection, lungs and/or broncho-alveolar lavage fluids obtained from wild-type (WT) (white bars) and ChemR23^−/−^ mice (black bars) were assessed for cytokine transcripts or proteins. (**A**) Lung IFN-α and IFN-β transcripts were assayed by qRT-PCR (upper panels). IFN-α levels were also determined by ELISA in lung homogenates (lower left panel) and broncho-alveolar lavage fluids (lower right panel). (**B**) Chemokine (KC/CXCL1) and cytokines (IFN-γ, TNF-α, IL-6, IL-12p40 and IL-17) levels were determined by ELISA in lung homogenates. Data are the mean ± SEM for groups of seven animals and are representative of three independent experiments. *, p<0.05; **, p<0.01.

We next investigated if the differences in clinical outcome and viral clearance could be explained in part by differences in the production of cytokines involved in anti-viral defenses. Various cytokines were tested by qRT-PCR and/or ELISA in basal conditions and at days 6, 8 and 10 post-infection. Most cytokines peaked at day 8 post-infection and decreased by day 10 (Figure 4 and Figure S2). Type I interferons, including IFN-α and -β, are known to play a central role in anti-viral immunity [18]. We first assayed IFN-α and -β transcripts in lung by qRT-PCR (Figure 4A). In contrast to WT mice, IFN-α and –β transcript levels increased only slightly in ChemR23^−/−^ mice and remained respectively 5-fold and 4-fold lower than in WT mice at day 8 post-infection (p<0.05). We confirmed by ELISA the lower levels of IFN-α in ChemR23^−/−^ compared to WT mice in lung homogenates (49±16 versus 158±51 pg/ml respectively; p<0.05), and BAL fluids (91±26 versus 239±46 pg/ml respectively; p<0.01) at day 8 post-infection. IFN-γ production was also assessed by qRT-PCR in lung, and using ELISA in lung, serum and BAL (Figure 4B and Figure S2). The levels peaked at day 8 post-infection, with no significant difference between ChemR23 KO and WT mice. Interleukin (IL)-12 (or IL-12p70), another important cytokine regulating the effector functions of lymphocytes, is composed of two subunits, p40 and p35. The IL-12 p40 subunit was investigated in lung, and significantly higher levels were found in WT mice than in ChemR23^−/−^ mice at days 8 and 10 post-infection, both for the protein (2.50±0.16 versus 1.40±0.30 ng/ml respectively at day 8, 1.30±0.20 and 0.50±0.03 ng/ml at day 10; p<0.05) (Figure 4B), and the transcript (∼3-fold difference; p<0.05) (Figure S2). As IL-12 p40 subunit is also shared by IL-23, a heterodimeric cytokine promoting the synthesis of IL-17, IL-17 synthesis was assessed in lung homogenates by ELISA and qRT-PCR, but no differences were observed between WT and ChemR23^−/−^ mice (Figure S2). IL-12 p70 was assayed by ELISA and a cytometric bead array-based immunoassay, but remained undetectable in BAL fluids and lung homogenates (data not shown). ChemR23^−/−^ mice displayed higher levels of IL-6 than WT mice at day 8 and the difference became significant at day 9 (1447±664 versus 288±38 pg/ml respectively, p<0.01) and day 10 post-infection (1301±328 versus 454±124 pg/ml respectively, p<0.05). Although all these cytokines were increased during the acute phase of the disease, no differences were observed between KO and WT mice for lung TNF-α, IL-1β, IL-5, IL-10, IL-13, and IL-17 (Figure 4B and Figure S2). Moreover, as neutrophils are massively recruited during PVM infection, KC (CXCL1), a major chemokine for neutrophils in mouse, was measured in lung homogenates. KC levels increased to a peak value by day 8 post-infection and remained significantly higher in ChemR23^−/−^ mice compared to WT mice at day 9 (86.4±20.5 versus 28.3±2.2 pg/ml respectively, p<0.01) and day 10 post-infection (50.3±5.4 versus 26.5±3.6 pg/ml respectively, p<0.01) (Figure 4B).

### ChemR23 deficiency results in a lower recruitment of plasmacytoid dendritic cells

**Figure 5:**
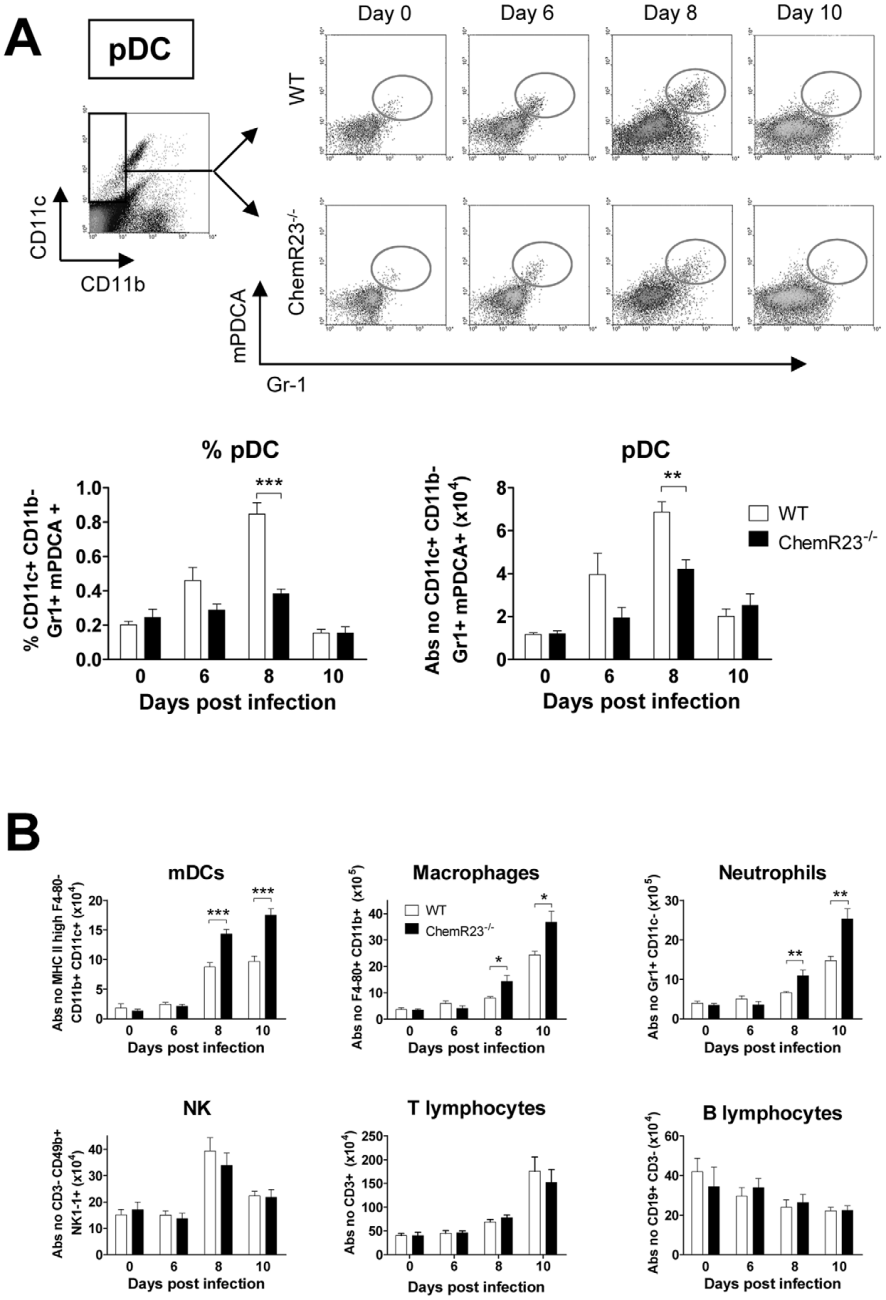
Plasmacytoid dendritic cell recruitment to the lung is less efficient in ChemR23^−/−^ mice during PVM infection. Inflammatory cell subsets were assessed by flow cytometry in lung cell suspensions obtained from wild-type (WT) (white bars) and ChemR23^−/−^ mice (black bars) either naive or 6, 8, and 10 days after PVM inoculation. (**A**) Plasmacytoid dendritic cells (pDC) were determined as the Gr-1^+^ mPDCA^+^ population after gating on CD11b^−^ CD11c^+^ cells (upper panels). The lower panels display respectively the percentage and absolute numbers of pDCs. (**B**) Absolute numbers of myeloid dendritic cells (mDC, CD11c^+^ CD11b^+^ MHC II^high^ F4-80^−^ cells), lung macrophages (F4-80^+^ MHC II^low^ CD11b^+^), neutrophils (Gr1^+^ CD11b^+^ CD11c^−^), NK cells (NK1-1^+^ CD3^−^), T (CD19^−^ CD3^+^) and B (CD19^+^ CD3^−^) lymphocytes at selected time points after infection. Data are the mean ± SEM for groups of at least five animals and are representative of at least three independent experiments. *, p<0.05; **, p<0.01; ***, p<0.001.

**Figure 6:**
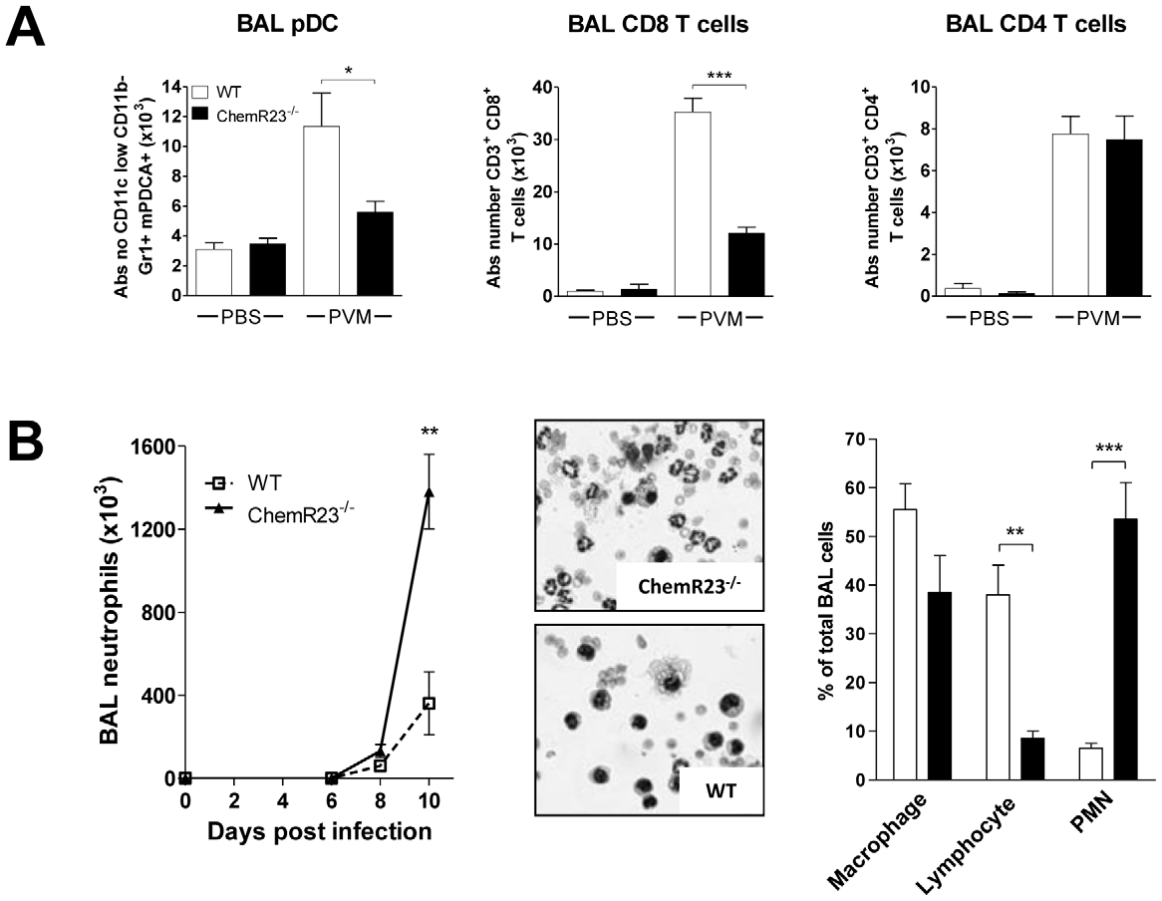
Decreased recruitment of pDCs and CD8^+^ T lymphocytes and higher recruitment of neutrophils to the lung of infected ChemR23-deficient mice. (**A**) Absolute numbers of pDCs, CD8^+^ and CD4^+^ T lymphocytes determined by flow cytometry in BAL fluids obtained from wild-type (WT) (white bars) and ChemR23^−/−^ mice (black bars), either non-infected or at day 8 post-infection. (**B**) Neutrophil counts were determined in BAL fluids at different time points post-infection in WT and ChemR23^−/−^ mice (left panel). Hematoxylin-eosin staining as well as the relative cell counts performed on cytospin preparations (middle and right panels) from BAL fluids obtained from wild-type (white bars) and ChemR23^−/−^ mice (black bars) at day 10 post-infection are displayed. Data are the mean ± SEM for groups of at least five animals. *, p<0.05; **, p<0.01; ***, p<0.001.

To investigate the different leukocyte populations recruited during the viral infection, cell suspensions were obtained from digested lungs at different time points after PVM inoculation and assessed by flow cytometry. As ChemR23 is highly expressed by pDCs and is one of the few functional chemoattractant receptors on these cells, the percentage and the absolute number of pDCs were determined (Figure 5A). PDCs were identified as Gr-1^+^ mPDCA^+^ cells after a first gating on the CD11b^−^ CD11c^+^ population. The number of pDCs in uninfected ChemR23^−/−^ mice did not differ from that in WT mice. A peak of pDCs was reached at day 8 post-infection with, in WT mice, a 5.8-fold increase from 11.7±0.8×10^3^ pDCs in basal conditions to 68.5±4.7×10^3^ pDCs per lung. In ChemR23^−/−^ mice however, the increase in pDCs was significantly lower than in WT mice (Figure 5A). At day 8 post-infection, pDCs represented, respectively in ChemR23^−/−^ and WT mice, 0.38±0.03% and 0.85±0.06% of the total cell number (p<0.001). The corresponding absolute numbers were respectively 42.0±4.4×10^3^ and 68.5±4.7×10^3^ pDCs per lung (p<0.01). Myeloid dendritic cells (mDCs) were identified as CD11c^+^ CD11b^+^ cells after a first gating on the MHC-II^high^ F4-80^−^ population (Figure 5B). MDCs also increased during viral challenge but, in contrast to pDCs, higher values were found in ChemR23^−/−^ than in WT mice. Indeed, at day 8 post-infection, 143±8×10^3^ mDCs were counted in ChemR23^−/−^ mice and 88±7×10^3^ cells in WT mice (p<0.001). This difference increased at day 10 with 175±13×10^3^ mDCs in ChemR23^−/−^ and 97±9×10^3^ mDCs in WT mice. Lung macrophages were defined as F4-80^+^ CD11b^+^ CD11c^−^ cells and neutrophils as Gr1^+^ CD11b^+^ CD11c^−^ cells. The number of macrophages and neutrophils increased from day 6 to day 10 post-infection, reaching respectively 6.5- and 3.8-fold basal values (Figure 5B). In ChemR23^−/−^ mice, macrophages and neutrophils were significantly higher than in WT mice at days 8 and 10 post-infection. At day 10, macrophage counts were respectively 3.6±0.4×10^6^ and 2.4±0.1×10^6^ cells in ChemR23^−/−^ and WT mice (p<0.05), and neutrophil counts were respectively 2.5±0.3×10^6^ and 1.5±0.1×10^6^ cells (p<0.01). No differences were observed between ChemR23^−/−^ and WT mice for NK cells (NK1-1^+^ CD3^−^), T cells (CD19^−^ CD3^+^) and B cells (CD19^+^ CD3^−^) (Figure 5B). Flow cytometry analysis was also performed on BAL fluids obtained at day 8 post-infection. Compared to uninfected mice, a significant increase in pDCs was observed in the WT group whereas this increase was not significant in ChemR23-deficient mice. In ChemR23^−/−^ mice, pDC counts reached only 50% of the values observed in WT mice (5.6±0.7×10^3^ versus 11.4±2.2×10^3^, p<0.05) (Figure 6A). As pDCs play an important role in anti-viral immunity through their ability to synthesize type I IFN that promote the cytotoxic response, the number of CD8^+^ cytotoxic T cells was determined in BAL fluids. Lower CD8^+^ T cell counts were found in ChemR23 KO mice (35.2±2.6×10^3^ versus 12.1±1.1×10^3^ for infected WT and KO mice respectively; p<0.001). No difference was observed for CD4^+^ T lymphocytes (7.7±0.8×10^3^ versus 7.5±1.1×10^3^ for WT and KO mice respectively; p>0.05) (Figure 6A). Whereas neutrophil counts in BAL fluid did not differ between WT and KO mice at day 8 post-infection, a major difference was seen at day 10. Indeed, neutrophils represented the main population in infected ChemR23^−/−^ mice but not in WT mice (respectively 53.5±7.6% and 6.5±1.1%; p<0.001). Moreover, the phenotype of BAL neutrophils in ChemR23 KO and WT mice was evaluated at days 10 and 14 post-infection, using flow cytometry. No difference was seen in the neutrophil population (F4/80^−^, Ly-6G^+^, CD11b^+^), in terms of FAS and ICAM-1 expression, which characterize the antitumoral/pro-inflammatory “N1” subset. The phenotype of BAL macrophages (F4/80^+^, Ly-6G^−^, CD11b^+^ cells) was also evaluated by flow cytometry at days 10 and 14 post-infection and no difference between ChemR23 KO and WT mice were seen for CD209a (DC-SIGN) expression as a marker of the alternatively activated and anti-inflammatory “M2” subset (data not shown). Altogether, these results do not support the recruitment of different subsets of neutrophils or macrophages to the lungs of infected ChemR23 KO and WT mice. The myeloperoxydase activity was also higher in ChemR23^−/−^ mice than in WT mice (Figure S3). In contrast, lymphocytes were the main population in WT mice at day 10 post-infection (38.0±6.1% versus 8.5±1.5% for WT and ChemR23^−/−^ respectively; p<0.01). No significant differences were observed for cells categorized as macrophages on the basis of their appearance on cytospin preparations (Figure 6B).

### Increased immunopathology and mortality rate in ChemR23^−/−^ mice are not due to defective pDC recruitment

**Figure 7:**
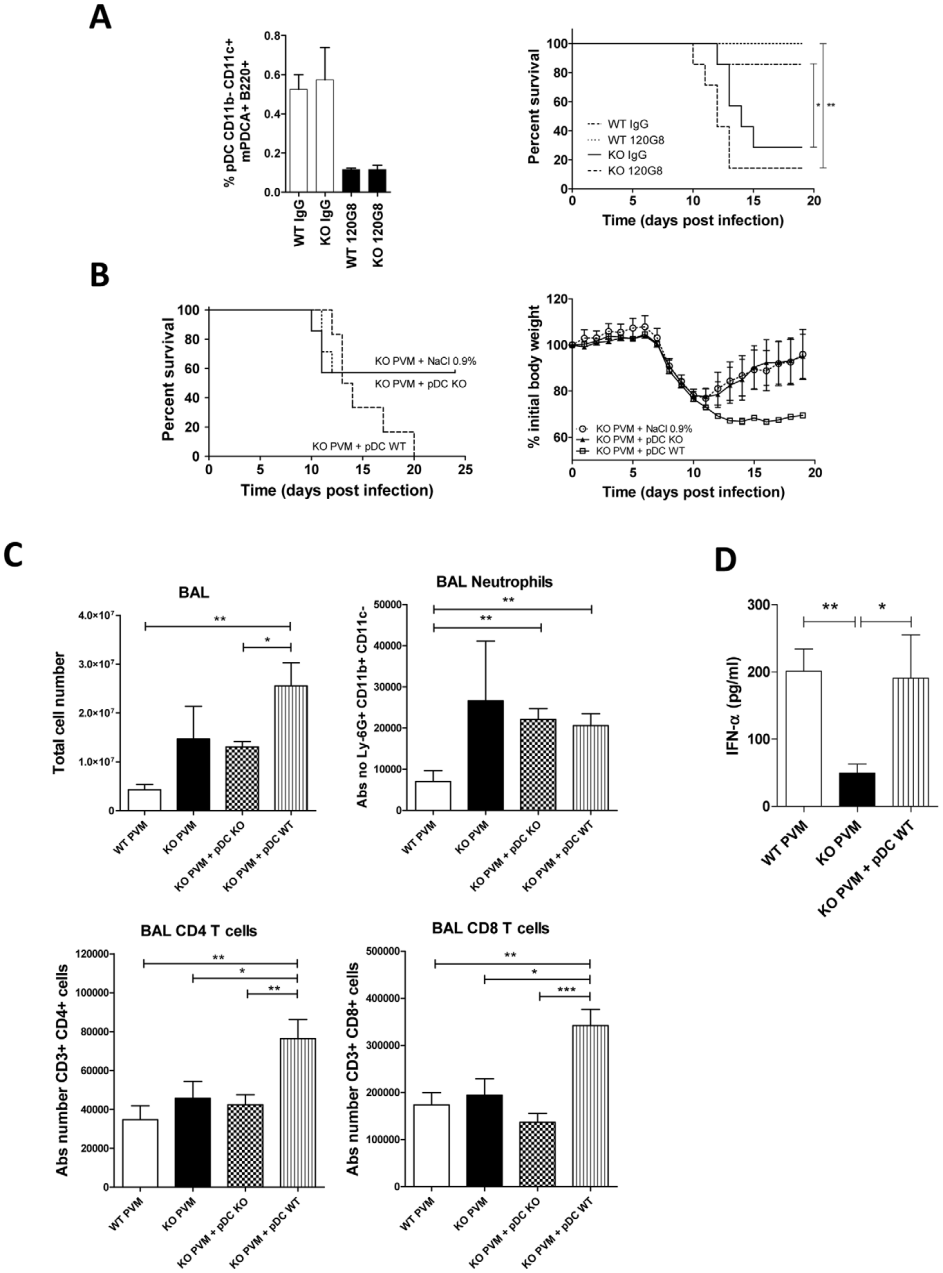
Increased pathogenicity of PVM in ChemR23^−/−^ mice is not due to the defective pDC recruitment. (**A**) Depletion of pDCs was achieved by using the 120G8 monoclonal antibody in wild-type (WT) and ChemR23^−/−^ (KO) mice infected by PVM. Depletion efficacy was assessed by flow cytometry analysis of pDCs (CD11b^−^ CD11c^+^ B220^+^ mPDCA^+^) among spleen cells collected at day 8 post-infection (left panel). Survival was recorded for infected WT and ChemR23^−/−^ mice, depleted or not in pDCs (n = 7 per group) (right panel). (**B**) 10^6^ pDCs from WT or ChemR23^−/−^ mice were transferred intravenously into knock-out mice at the time of infection with PVM. Knock-out mice receiving a saline solution were used as controls. Survival (left panel) and body weight (right panel) were monitored (n = 6 per group). (**C**) 10^6^ pDCs from WT or ChemR23^−/−^ mice were transferred intravenously into ChemR23^−/−^ mice at the time of infection (n = 7 per group). Infected wild-type and knock-out mice receiving a saline solution were used as controls (n = 5 per group). Mice were sacrificed 14 days after infection and the cell populations were analyzed in BAL fluids using flow cytometry. Histograms show the mean ± SEM for total cell numbers and absolute numbers of neutrophils, CD3^+^CD4^+^ and CD3^+^CD8^+^ T lymphocytes. (**D**) 10^6^ pDCs from WT mice were transferred intravenously into ChemR23^−/−^ mice at the time of infection. Infected wild-type and knock-out mice were used as controls. IFN-α levels were detected 9 days after infection in BAL fluids using ELISA (n = 5 per group). *, p<0.05; **, p<0.01, ***, p<0.001.

Our data suggest the contribution of ChemR23 in pDC recruitment, and impaired pDC recruitment in ChemR23^−/−^ mice might in turn explain lower synthesis of type I interferons and delayed viral clearance. In order to test the potential link between this cascade of events and the increased morbidity/mortality observed in KO mice, we performed pDC depletion and adoptive transfer experiments. Depletion of pDCs was achieved by intraperitoneal injections of the 120G8 monoclonal antibody, starting before PVM inoculation. Staining of spleen cells obtained at day 9 post-infection confirmed the efficacy of the protocol. With the exception of B cells, which were slightly but significantly (p<0.05) decreased in the depleted WT group, no effect of the depleting antibody was observed on other subsets of inflammatory cells, including mDCs, macrophages and T cells (Figure 7A and Figure S4). Following PVM infection, a higher mortality rate (55%) was observed for ChemR23^−/−^ mice (Figure 7A), in line with our previous experiments. However, pDC depletion in WT mice did not increase their mortality rate, compared to WT mice treated with a control IgG. Furthermore, pDC depletion did not reduce the difference in mortality rate between WT and ChemR23^−/−^ mice.

Adoptive transfer of pDCs was also performed. PDCs were purified from the spleen of WT and ChemR23^−/−^ mice overexpressing Flt3 ligand. The generated pDCs were tested for their phenotype and functionality, and no difference in their ability to synthesize cytokines (IFN-α and IL12p40) in response to CpG stimulation was detected *in vitro* (Figure S5). Moreover, pDCs from WT mice were able to migrate in response to chemerin with a typical bell-shaped curve culminating for concentrations around 1 nM, whereas pDCs from KO mice failed to migrate in response to chemerin (Figure S6). If defective pDC recruitment was causal in the differences observed between WT and ChemR23^−/−^ mice, we expected that adoptive transfer of ChemR23-expressing pDCs to knock-out mice would reduce the mortality and weight loss in this group, as compared to knock-out mice receiving either pDCs from KO mice or a saline solution. Interestingly, these experiments showed the opposite effect (Figure 7B). ChemR23^−/−^ mice receiving a saline solution or pDCs from knock-out mice presented similar weight and survival curves, whereas ChemR23^−/−^ mice receiving pDCs from WT mice displayed higher weight loss and mortality rate. We also observed more severe clinical signs in these animals, particularly reduced locomotor activity and increased crackles. These observations were complemented by the analysis of cells in BAL fluids, using flow cytometry. We showed that ChemR23^−/−^ mice receiving pDCs from WT mice displayed an increase in the number of cells in BAL, as compared to ChemR23^−/−^ mice receiving pDCs from KO mice, 14 days after infection/adoptive transfer of pDCs. The cells were mainly CD3^+^CD4^+^ and CD3^+^CD8^+^ T lymphocytes, while neutrophils were low at that time (Figure 7C). In these settings, pDCs, NK cells and macrophages were low (data not shown). Analysis of the cytokine levels in BAL fluid showed a sustained production of IL-6 compared to WT mice and higher levels of IFN-γ compared to WT and KO mice (Figure S7), while levels of KC were unchanged and no IL-10 was detected at this time-point (data not shown). Finally, the restoration of the IFN-α production was detected using ELISA in the BAL fluid of ChemR23^−/−^ mice receiving pDCs from WT mice, as compared with levels in the BAL fluid of WT mice (Figure 7D).

### The inflammatory phenotype of KO mice is not due to the lack of ChemR23 expression by leukocytes

**Figure 8:**
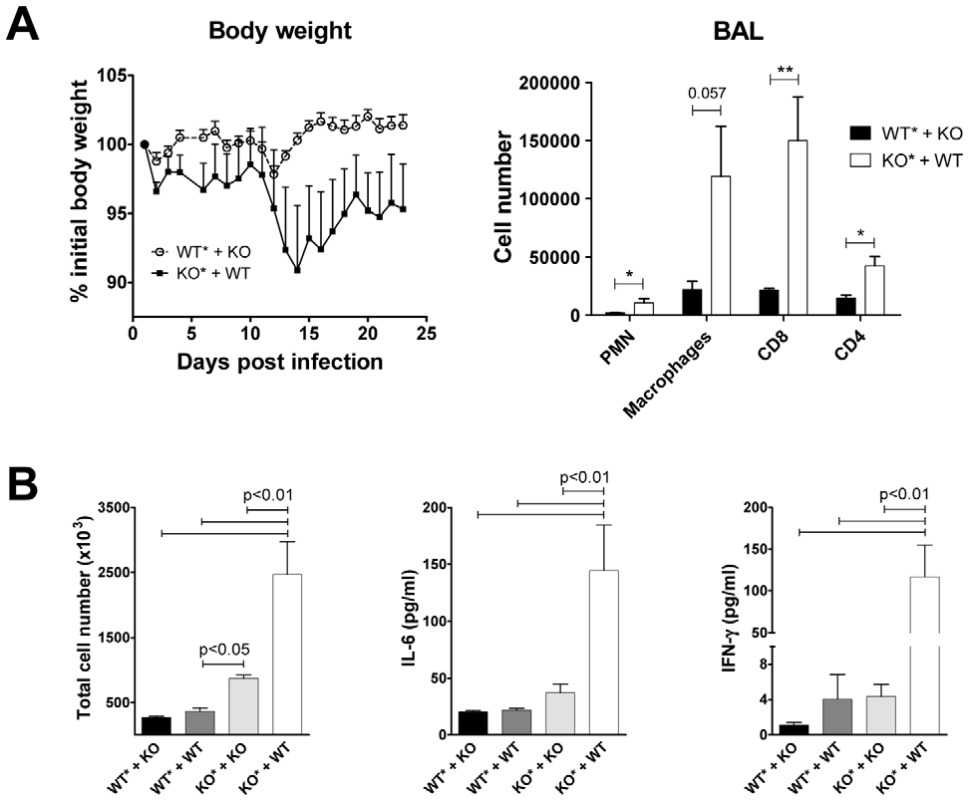
Transfer of bone marrow from wild-type mice does not protect ChemR23-deficient mice from PVM infection. (**A**) Irradiated (*) wild-type mice reconstituted with ChemR23^−/−^ bone marrow (WT*+KO), as well as irradiated KO mice reconstituted with bone marrow from WT mice (KO*+WT), were infected by PVM (1000 PFUs) and monitored for weight loss (left panel). 23 days post-infection, mice were sacrificed and inflammatory cells were counted in BAL fluids (right panel). (**B**) BAL fluids were also harvested at day 14 post-infection in a similar experiment including two additional groups, namely WT and KO mice reconstituted respectively with WT and KO bone marrow (WT*+WT, KO*+KO, respectively). Total cell numbers (left panel) as well as cytokine levels (IL-6 and IFN-γ, middle and right panels) were determined in these samples. Data are the mean ± SEM for groups of at least five animals. *, p<0.05; **, p<0.01.

As lower pDC recruitment does not explain the higher immunopathology observed in ChemR23 KO mice, we further characterized the role of ChemR23 expression by leukocytes versus non leukocytic cells in PVM infection pathogenesis. To address this question, irradiated WT and KO mice were reconstituted with bone marrow (BM) cells harvested from KO and WT mice respectively, in order to generate WT mice having ChemR23-deficient leukocytes and conversely. Chimerism was assessed by flow cytometry 6 weeks post-irradiation and we observed that more than 90 percent of CD45^+^ spleen cells resulted from the graft (GFP-positive cells; 94.0±1.5%; mean ± SEM for n = 6 independent experiments). Irradiated and reconstituted mice presented a lower susceptibility to PVM infection than sex- and age-matched non-irradiated mice (data not shown). This observation is in line with previously published data showing lower asbestos-induced lung inflammation after myelo-ablative irradiation and BM transplantation [37]. As a result, all mice survived the PVM challenge and a relatively mild weight loss was observed. As shown in Figure 8, KO mice reconstituted with WT leukocytes presented higher weight loss and displayed more severe illness signs than WT mice reconstituted with KO leukocytes. At the end of the observation period, mice were sacrificed and higher numbers of neutrophils, macrophages, CD8^+^ and CD4^+^ T cells were recovered in BAL fluids of KO mice reconstituted with WT leukocytes (Figure 8A). These observations were confirmed in other experiments in which BAL fluids were harvested earlier (day 14 post-infection), and including additional groups of WT and KO mice reconstituted respectively with WT and KO mice (Figure 8B). Higher numbers of inflammatory leukocytes were found in BAL fluids of KO mice grafted with BM from KO mice, than in the two WT groups. However, as in the first experiment, KO mice reconstituted with WT leukocytes presented much higher BAL cell counts, as well as IL-6 and IFN-γ levels.

## Discussion

In this study, we investigated the role of the chemerin-ChemR23 system in a mouse model of viral pneumonia. Wild-type and ChemR23 knock-out mice were infected by PVM, the mouse counterpart of human RSV. The two viruses are closely related and evoke similar immune responses. The use of the natural mouse pathogen PVM was however preferred, because it replicates efficiently following a minimal virus inoculum, and recapitulates many of the clinical and pathologic features of the most severe forms of RSV infection in human infants. By contrast, mice are relatively resistant to infection by human RSV [10].

We demonstrated that ChemR23^−/−^ mice develop a more severe inflammatory status than wild-type mice, resulting in a significant increase in morbidity and mortality rate. ChemR23^−/−^ mice presented a higher recruitment of neutrophils and macrophages, higher myeloperoxydase activity, extended macroscopic and microscopic lesions, increased synthesis of pro-inflammatory cytokines (*e.g.* IL-6) and chemokines (*e.g.* CXCL-1/KC) and higher levels of bioactive chemerin in the lung. As a direct consequence, ChemR23-invalidated mice showed a more severe respiratory dysfunction (restrictive syndrome), evaluated by double chamber whole body plethysmography. We demonstrated a lower recruitment of pDCs to the lung of ChemR23^−/−^ mice, despite a 100-fold higher viral load. In line with the high expression of ChemR23 on immature plasmacytoid dendritic cells and the chemoattractant activity of chemerin on pDCs *in vitro*, our data strongly suggest a major role of the chemerin/ChemR23 system in the recruitment of pDCs *in vivo*. This observation correlates with other studies reporting a link between chemerin expression and the recruitment of pDCs and NK cells in human inflammatory diseases of the skin [27], [31], [38], [39]. The recruitment to lung of other ChemR23-bearing cells, including macrophages, myeloid dendritic cells and NK cells was apparently not affected in ChemR23^−/−^ mice. This could be explained by the lower expression of ChemR23 on these cells and the redundancy with other chemoattractant molecules able to recruit these cell populations [31], [40], [41]. In this context, the higher number of mDCs and macrophages observed in lungs of infected ChemR23^−/−^ mice might be explained by the higher inflammatory state and the higher production of chemokines acting independently of the chemerin/ChemR23 system. Chemerin levels were found to be increased in lung and airways during viral infection, reaching higher values in ChemR23^−/−^ mice than in controls. As prochemerin transcript levels did not change during the course of the disease, the strong upregulation of chemerin in lung likely results from exudation of circulating prochemerin. The observation of bioactivity on ChemR23-expressing cells demonstrates that prochemerin was also processed to its active forms, presumably in part by neutrophil proteases. Chemerin and neutrophil recruitment increase indeed in parallel during the course of infection. The higher levels of chemerin immunoreactivity and bioactivity in ChemR23^−/−^ mice may be explained in part by the stronger inflammatory response, resulting in more exudation and more efficient activation of prochemerin by proteases released by the higher number of neutrophils. However, basal immunoreactive chemerin levels are also increased in uninfected animals. Increased (pro)chemerin levels may therefore also result from defective removal of the protein from extracellular fluids, resulting from the lack of receptors (ChemR23) able to bind and internalize chemerin. This hypothesis of chemerin scavenging by ChemR23-positive cells is supported by similar observations made on mice invalidated for chemokine receptors in which the circulating levels of the corresponding chemokine ligands were found to be elevated, as described for the CCL2/CCR2 axis [42].

PDCs play an important role in anti-viral immunity by their capacity to secrete large amounts of type I IFNs (including IFN-α and -β) and induce effector CD8^+^ T-cell response upon viral infection [13]. Type I IFNs have pleiotropic anti-viral functions: they increase the resistance of non-infected cells to viral infection, inhibit viral gene transcription, induce apoptosis of infected cells, induce B cell differentiation into antibody-secreting plasma cells, promote the cytotoxic activity of NK and CD8^+^ T cells, promote survival and proliferation of CD8^+^ T cells, as well as differentiation and maturation of DCs [18], [43]–[46]. As a result, type I IFNs promote an efficient acquired immune response and facilitate viral clearance in PVM-induced mouse pneumonia models [7]. In PVM-infected ChemR23^−/−^ mice, it is therefore tempting to link the defective pDC recruitment to the lower synthesis of type I IFNs, lower acquired immune response (low CD8^+^ T cell counts and IL-12p40 production), and delayed viral clearance. In this context, parameters unaffected (IFN-γ production) or displaying mild differences (anti-PVM antibodies production) must be interpreted taking into account a 100-fold higher viral load in ChemR23 KO mice. In our study, no difference in viral loads were observed before day 8 post-infection, suggesting that intrinsic viral replication of PVM was not affected by ChemR23 deficiency. Moreover, replication of PVM was reported in type I and II pneumocytes, alveolar macrophages, eosinophils, and bronchiolar epithelial cells, which are not described to express ChemR23 [34]–[36], [47 and unpublished observations].

Therefore, to investigate whether lower recruitment of pDCs in PVM-infected ChemR23^−/−^ mice might explain the excessive morbidity, pDC depletion and adoptive transfer experiments were performed. In depletion experiments, mortality rates were essentially unchanged both in WT and ChemR23^−/−^ mice, suggesting that pDCs do not play a major role in the poor clinical outcome of PVM-infected ChemR23^−/−^ mice. Moreover, adoptive transfer of ChemR23-expressing pDCs to KO mice enhanced clinical symptoms and decreased survival. Interestingly, previous studies have shown that C57BL/6 mice lacking type I IFN receptor (IFN-αβR^−/−^) or functional TCR exhibit defective acquired response and higher PVM titers, but milder histological lesions, decreased PMN recruitment and lower mortality rate [7], [8]. Taken together, these observations strongly suggest that the extent of lung tissue damage is not directly related to the viral load, but rather to the efficiency of the anti-PVM immune response, including the acquired Th1 component. In this context, improving the immune response by transferring ChemR23-expressing pDCs may indeed lead to a more severe lung disease. In other models however, pDC depletion resulted in an enhancement of the inflammatory state. In pDC-depleted BALB/c mice infected by human RSV, a higher Th2 response was observed, which was found to be IFN-independent [21]. PDC depletion in BALB/c mice also resulted in enhanced airway inflammation and eosinophilia in asthma models, whereas adoptive transfer of pDCs suppressed inflammation and the Th2 response [25]. This immunomodulatory role of pDCs on Th2 responses is type I IFN-independent and mediated by their ability to induce regulatory T lymphocytes and/or to suppress effector T cell generation [14], [23]–[25]. The influence of pDCs may therefore affect the inflammatory state positively or negatively, according to the specific model considered and the main immune responses triggered in this model.

As defective pDC recruitment did not appear to underlie the more severe clinical outcome observed in ChemR23 KO mice, we hypothesized that these mice might have lost a direct anti-inflammatory pathway involving ChemR23. This hypothesis is indeed supported by previous results from our group, showing a ChemR23-dependent anti-inflammatory role of chemerin in a LPS-induced acute lung inflammation model [28]. To assess whether this anti-inflammatory effect is mediated by ChemR23-expressing leukocytes or other cell populations, chimeric mice were generated following lethal irradiation and BM adoptive transfer. These experiments showed that mice lacking ChemR23 only in BM-derived cells behaved essentially as wild-type animals, while restoring ChemR23-expression in leukocyte populations did not protect KO mice from excessive inflammatory response to PVM infection. It appears therefore that the chemerin/ChemR23 system has anti-inflammatory properties on a non-leukocytic cell population, which is presently not identified. Lung endothelial cells might constitute a candidate cell population, since ChemR23 expression was recently described in human endothelial cells [48], [49]. Chemerin might therefore regulate trafficking of inflammatory cells such as PMNs, by modulating the release of chemokines by endothelial cells, or their interactions with leukocytes. Finally, our chimera experiments showed that mice expressing ChemR23 exclusively in leukocytes displayed the most severe inflammatory phenotype, with the highest levels of IFN-γ and CD8 T cells in BAL fluids. Similar results were obtained following adoptive transfer of ChemR23-expressing pDCs in KO mice. These observations suggest that ChemR23-expressing leukocytes, among which pDCs, contribute significantly to the innate and acquired cytotoxic responses and the resulting inflammatory insult to lung parenchyma.

In conclusion, ChemR23-deficient mice are more susceptible to PVM infection. The recruitment of pDCs is impaired in these mice, resulting in a reduction of type I IFN synthesis and delayed viral clearance. However, the stronger inflammatory status and the resulting higher morbidity and mortality, are not the consequence of impaired pDC recruitment. Chemerin appears therefore to have anti-inflammatory properties, by acting on ChemR23 expressed by non-leukocytic cells, thereby dampening the inflammatory response promoted by the viral infection. Further analyses are needed to determine the precise underlying mechanisms and cell types involved in these processes.

## Materials and Methods

### Ethics statement

This study was carried out in strict accordance with the national, european (EU Directives 86/609/EEC) and international guidelines in use at the Université Libre de Bruxelles. All procedures were reviewed and approved by the ethical committee (Commission d'Ethique du Bien-Etre Animal, CEBEA) of the Université Libre de Bruxelles (Permit Number: 222N and 341N). All efforts were made to minimize suffering.

### Mice

Eight to twelve weeks-old C57BL/6 mice (Harlan Netherlands) were used throughout these studies. ChemR23-deficient mice (KO) were obtained from Deltagen (CA, USA) through Euroscreen S.A. (Brussels, Belgium). They were backcrossed for 12 generations into the C57BL/6 background in a specific pathogen free environment. Wild-type littermates from F_1_ matings were used as controls.

### PVM-induced viral pneumonia model

The J3666 strain of PVM (initially provided by A.J. Easton) was passed in BALB/c mice and grown once onto BSC-1 cells to produce the viral stock. Randomly selected aliquots of the stock yielded highly reproducible titers on BSC-1 cells, amounting to 1×10^6^ PFU/ml. Mice were inoculated under brief anaesthesia (ketamine, Pfizer, 50 mg/kg, and xylazine, Bayer, 10 mg/kg, i.p.) by intranasal instillation of 50 µl of a viral suspension containing 1000 PFU and 1% BSA in PBS. Control mice were inoculated with PBS containing 1% BSA. At selected time intervals (6, 8 and 10 days post-infection), groups of minimum 6 mice were sacrificed with sodium thiopental (5 mg/animal, i.p.). In some experiments, broncho-alveolar lavage fluids were obtained by flushing the lungs with sterile 0.9% NaCl, and differential cell counts were performed on cytospin preparations after Diff-Quick staining (Dade Behring).

### Whole body plethysmography

Lung function was assessed in groups of five mice using a whole body double chamber plethysmograph (Buxco) and IOX software (EMKA Technologies) as described previously [50]. Briefly, at selected time points (before inoculation and 1, 3, 5, 6, 7 and 8 days post-inoculation), awake mice were placed between two compartments (nasal and thoraco-abdominal), in which flow variations were recorded during 5 minutes and used to determine the following parameters: tidal volume (TV); expiratory balance; RT/Te, in which RT is the relaxation time determined as the time needed to expire 64% of the inspired volume and Te the expiratory time; end expiratory pause (EEP), Te – RT; enhanced pause (Penh); specific airway resistance (sRaw).

### Lung histological analysis and anti-PVM immunostaining

At day 9 post-infection, left lungs were insufflated with 500 µl of 4% paraformaldehyde, and embedded in paraffin. Sections (5 µm) were stained with haematoxylin and eosin and assessed by light microscopy. Paraffin embedded lung sections (5 µM) were also used for PVM antigen immunofluorescence staining using an anti-PVM antiserum (dilution 1/100, rabbit) and an Alexa Fluor 488-conjugated anti-rabbit IgG antibody (dilution 1/1000, Invitrogen). Images were captured with an Axioplan 2 imaging fluorescence microscope, equipped with a Diagnostic Spot digital camera and analyzed by the Spot Advanced Soft Imaging System and Adobe Photoshop 7.0. The lenses used were 20×/0.5, ∞/0.17; 40×/0.75, ∞/0.17 and 100×/1.30, ∞/0.17.

### Viral titration

At selected time points, viral titers were determined by standard plaque assay as previously described [34]. Briefly, the right lung was homogenized in PBS containing 1% BSA and successive 10-fold dilutions of the supernatant were used to infect BSC-1 cell cultures. The viral suspensions were left to adsorb for 3 h at 31°C, after which the cell monolayers were covered with 1 ml of 0.6% agarose in MEM containing 2% FBS. After incubation at 31°C for 12 days, the agar overlay was removed, the remaining cells were stained with crystal violet, and titers were determined by counting the number of plaque forming units (PFUs).

### Detection of PVM antibodies in serum

The serum was evaluated for anti-PVM antibodies on days 9, 10, and 11 post-inoculation and compared to serum obtained from control mice using the SMART-M12 kit (Biotech Trading Partners).

### Quantitative RT-PCR

Lung total RNA was extracted and purified using the RNeasy Mini Kit (Qiagen). After DNase I treatment (Ambion), samples were reverse transcribed into cDNA using random hexamers (Roche) as primers and the Superscript II polymerase (Invitrogen). RT-PCR products were analyzed by quantitative real-time RT-PCR. The sequence of primer pairs used for mouse chemerin, IFN-α, IFN-β, CANX, and YWHAZ is provided in Table S1. Raw data were normalized for each sample using YWHAZ and CANX gene expression as references.

### Cytokine assays

At selected time points, right lungs were homogenized in PBS and supernatants were assayed for TNF-α, IL-6, IFN-γ and KC/CXCL1 using cytometric bead array-based immunoassays (CBA Flex set, BD Biosciences), a dual-laser flow cytometer (FACSCalibur, BD Biosciences) and the FCAP Array software (BD Biosciences) for analysis, following the manufacturer's instructions. IL-12p40, IL-17, chemerin, and IFN-α were determined by ELISA in lung homogenates (R&D Systems, Abingdon, UK). Chemerin, as well as IL-6, TNF-α, IL-10, IFN-γ (BD Biosciences), IFN-α and KC (R&D Systems) were also measured in BAL fluids using ELISA according to the manufacturer's instructions.

### Biological activity of BAL chemerin

BAL fluids from two infected WT and ChemR23^−/−^ mice were pooled, filtered and loaded onto a C18 reverse phase column (2.1×250 mm; Vydac). Fractions from a 25–50% acetonitrile (CH_3_CN) gradient (0.5%/min) in 0.1% TFA were collected. The biological activity was next measured by a calcium-mobilizing assay based on the bioluminescence of aequorin, using CHO-K1 cells co-expressing mouse ChemR23, apoaequorin and G_α16_ [30]. CHO-K1 cells co-expressing only apoaequorin and G_α16_ were used as control. Fractions from infected WT mice were also concentrated and depleted of acetonitrile using a vacuum SpeedVac concentrator and then tested undiluted in the same assay. Results (expressed as luminescence units) were normalized to the response to 20 µM ATP. A dose-response curve for chemerin on ChemR23-positive CHO-K1 cells was also generated using 0.01 to 100 nM recombinant mouse chemerin (R&D Systems).

### Flow cytometry

Lungs were perfused with 10 ml PBS through the right ventricle, dissected, minced, and incubated with 2 mg/ml collagenase D and 0.02 mg/ml DNAse I (Roche) for 1 hour at 37°C. In some experiments, cell suspensions were obtained from BAL fluids as previously described. After lysis of red blood cells and blockade of non-specific binding to FcR with anti-CD16/CD32 monoclonal antibodies (mAbs), viable cells were counted by trypan blue exclusion. Cells were stained with mAbs directed against F4/80 (FITC or APC), CD11b (FITC or PerCp-Cy5.5), CD4 (FITC or Horizon V450), CD11c (PE or APC), I-A/I-E (PE), CD19 (PE), NK1-1 (PE), Gr-1 (PerCp-Cy5.5), Ly6-G (Alexa 700), CD8 (PerCp or Horizon V500), ICAM-1 (PE), CD95 (PE-Cy7), CD209a (biotinylated), Streptavidin-FITC, CD3 (APC), mPDCA (APC), and isotype controls (all from BD Biosciences except F4/80-FITC from AbD Serotec). All samples were analyzed using a dual-laser flow cytometer (FACSCalibur) using the CellQuest software (BD Biosciences) or a four-laser flow cytometer (FACS LSRFortessa) using FACS Diva software (BD Biosciences).

### Depletion of pDCs

Depletion of pDCs was performed in WT and ChemR23^−/−^ mice using a monoclonal antibody against pDCs (120G8, Dendritics). Mice received four i.p. injections of 125 µg 120G8, every other day, beginning the day before infection. Depletion efficacy and specificity was assessed by assaying cell populations in spleen. After mechanical disruption, isolated spleen cells were stained for pDCs, mDCs, macrophages, T and B cells using the following set of antibodies: B220 (FITC), CD11c (PE), CD11b (PerCp-Cy5.5), mPDCA (APC); or F4-80 (FITC), CD19 (PE), CD11b (PerCp-Cy5.5) and CD3 (APC).

### Adoptive transfer of pDCs

The generation, purification and transfer of isolated pDCs were performed as previously described [25]. Briefly, wild type and ChemR23^−/−^ mice were injected subcutaneously with 5×10^6^ melanoma B16 cells (syngeneic to C57BL/6 mice) expressing the dendritic cell growth factor FMS-like tyrosine kinase 3 ligand (Flt3-ligand) (kindly provided by M. Moser, ULB, Gosselies). Three weeks later, mice were sacrificed and their spleen mechanically disrupted. Released cells were then passed through a 70 µM cell strainer (BD Biosciences). Untouched pDCs from single-cell suspensions were then isolated by negative selection (Plasmacytoid Dendritic Cell Isolation Kit II, Miltenyi Biotec). Briefly, non-pDC cells were labelled with a cocktail of biotin-conjugated monoclonal antibodies and depleted by retention on a MACS column. The viability and purity of the pDC preparations were respectively over 95% and 90%, as assessed by flow cytometry following staining using respectively propidium iodide (PI) and mAbs against B220 (FITC), CD11c (PE), CD11b (PerCp-Cy5.5) and mPDCA (APC) (all from BD Biosciences). Thereafter, 10^6^ pDCs from WT mice in 0.9% NaCl were injected in the tail vein of ChemR23^−/−^ mice infected the same day by PVM. Weight loss and mortality rate were compared between this group (KO+pDC WT), infected ChemR23^−/−^ mice receiving the saline carrier (KO+0.9% NaCl), and infected ChemR23^−/−^ mice receiving 10^6^ pDCs purified from ChemR23^−/−^ mice (KO+pDC KO).

### Bone marrow transplantation and generation of chimeric mice

Chimeric mice were generated to discriminate between the roles of ChemR23 expressed by leukocytes or other cell types. For this purpose, 8 week old WT and ChemR23 KO mice received a lethal irradiation dose of 800 Rad TBI delivered by a ^137^Cesium irradiator. Twenty four hours later, irradiated mice were reconstituted by i.v. injection with 20×10^6^ syngeneic BM cells isolated from 6 to 8 week old WT or KO mice. BM cells were obtained by flushing BM from femurs and tibiae of donor mice. After 4 weeks, mice were infected and chimerism was assessed on spleen cells purified from mice reconstituted with BM cells from age-matched WT C57BL/6 mice expressing green fluorescent protein (GFP) (% GFP^+^ cells among CD45^+^ spleen cells).

### Statistical analysis

Significance was determined using Student's t test or one-way analysis of variance, using the Prism4 software (GraphPad). The Student-Newman-Keuls test was used for pairwise comparisons. Kaplan-Meier survival curves were compared using the logrank test. For all tests, p<0.05 was considered as significant.

### Accession numbers

(Source: http://www.ncbi.nlm.nih.gov) [Mus musculus]

Chemerin: 71660/NP082128; ChemR23/cmklr1: 14747/P97468; TNF-α: 21926/CAA68530; IL-10: 16153/NP034678; TGF-β1:21803/AAH13738; KC/CXCL-1: 14825/P12850; IL-6: 16193/P08505; IFN-α: 15962/P01572; IFN-β: 15977/P01575; IFN-γ: 15978/P01580; IL-5: 16191/P04401; IL-13: 16163/P20109; IL-17: 16171/Q62386; IL-12p40: 16160/P43432; IL-1β: 16176; CANX: 12330; YWHAZ: 22631.

## Supporting Information

Figure S1 **Assessment of respiratory functional parameters in PVM-infected wild-type and ChemR23^−/−^ mice using a whole-body double chamber plethysmograph.** Lung function was assessed using a double chamber plethysmograph. (**A**) Awake mice were placed between the two compartments (nasal and thoraco-abdominal) of the instrument, flow variations were recorded and the following functional parameters were determined: inspiratory time (Ti); expiratory time (Te); peak inspiratory flow (PIF); peak expiratory flow (PEF); tidal volume (TV); expiratory balance (RT/Te), in which RT is the relaxation time determined as the time needed to expire 64% of the inspired volume; end expiratory pause (EEP), Te - RT; and enhanced pause (Penh), ((TE/RT)-1)x(PEF/PIF) (E4). (**B**) Before and at selected time points post-infection, ChemR23^−/−^ (closed circles) and wild-type (WT) (open circles) mice were assessed for respiratory function. Whereas no significant changes were observed in WT mice, ChemR23^−/−^ mice displayed severe changes at days 7 and 8 post-infection, consistent with marked respiratory dysfunction and more specifically a restrictive syndrome. Along with a reduction in tidal volume, ChemR23^−/−^ mice displayed a 2.3-fold increase in end expiratory pause (p<0.05), a 3.4-fold increase in enhanced pause (Penh) (p<0.001), and a 65% reduction in expiratory balance (p<0.05). The displayed data are the mean ± SEM for groups of at least four animals, and are representative of two independent experiments. *, p<0.05; ***, p<0.001.
(TIF)

Figure S2 **Cytokinic profile in lung determined by ELISA and quantitative RT-PCR.** (**A**) Lung total RNA was extracted, purified and reverse transcribed into cDNA that was analyzed by quantitative real-time PCR. Sequences of primer pairs are displayed in Table S1. The data were normalized using two housekeeping genes (YWHAZ and CANX) as references, and reported to the corresponding transcript level in uninfected control mice. Assayed cytokines were TNF-α, IL-12p40, IL-10, IL-13, IL-17, TGF-β and IFN-γ. When gene expression was upregulated during the course of infection, a peak value was obtained at day 8 post-infection without significant differences between wild-type (WT) and ChemR23^−/−^ mice, except for IL-12p40 (∼3-fold higher values at days 8 and 10 post-infection in ChemR23^−/−^ mice; p<0.05). (**B**) IFN-γ in serum and BAL fluids, as well as IL-1β, IL-5 and IL-10 in lung homogenates were measured by ELISA in ChemR23^−/−^ and WT mice before and at various time points after PVM infection. No significant difference was observed for these cytokines. Data are the mean ± SEM for groups of at least five animals. *, p<0.05; **, p<0.01.
(TIF)

Figure S3 **Higher myeloperoxydase activity in the lung of infected ChemR23-deficient mice.** Myeloperoxydase (MPO) activity was assayed in lung homogenates from wild-type (WT) and ChemR23^−/−^ mice, 9 days after viral inoculation or PBS instillation. Cell pellets from homogenized lungs were resuspended in PBS containing 13.7 mM of hexadecyltrimethyl ammonium bromide (HTAB) and 5 mM EDTA. Following centrifugation, supernatants were harvested and diluted in Hanks' balanced salt solution (HBSS) containing 1 mM HTAB, 0.4 mM EDTA, 0.15 mM of *o*-dianisidine dihydrochloride solution and 0.56 mM of H_2_O_2_. After 15 minutes at 37°C, the reaction was stopped with 25 µl of 1% NaN_3_. The MPO activity was determined by measuring the absorbance at 460 nm against medium. No difference was observed between uninfected WT and ChemR23^−/−^ mice, whereas infected ChemR23^−/−^ mice presented a significantly higher MPO activity than infected WT mice. The data represent the mean ± SEM for groups of seven animals and are representative of three independent experiments. **, p<0.01.
(TIF)

Figure S4 **Assessment of the specificity of pDC depletion by the 120G8 antibody.** To investigate the role of pDCs in the stronger inflammatory response observed in infected ChemR23^−/−^ mice, pDC depletion experiments were performed. This was achieved by using a commercially available antibody (120G8). To assess the specificity of this depletion procedure, spleens from wild-type (WT) and ChemR23^−/−^ (KO) mice treated or not by the 120G8 antibody were obtained at day 8 post-infection. Cells were isolated and stained for myeloid dendritic cells (mDCs) (CD11c^+^ CD11b^+^ B220^−^ mPDCA^−^), macrophages (F4-80^+^ CD11b^+^ CD11c^−^), T (CD3^+^ CD19^−^ F4-80^−^) and B (CD19^+^ CD3^−^ F4-80^−^) lymphocytes. There was a mild but significant (p<0.05) decrease in the proportion of B cells in depleted WT (but not ChemR23^−/−^) mice, as compared to control IgG-treated mice. No effect of the depleting antibody was observed for mDCs, macrophages and T cells. Data are the mean ± SEM for groups of five animals. * = p<0.05.
(TIF)

Figure S5 **Production of IFN-α and IL-12p40 is not impaired in ChemR23^−/−^ pDCs.** The capacity of pDCs purified from wild-type (WT) and ChemR23^−/−^ mice to synthesize and secrete type I IFNs and IL-12p40 subunit was studied following stimulation by CpG (a TLR9 agonist). pDCs were prepared from spleen cells as described, resuspended at a density of 10^5^ cells/ml, stimulated in vitro by CpG (3 µg/ml), and the production of IFN-α and IL-12p40 was assayed by ELISA on the supernatant collected 24 hours later. Experiments were performed in triplicates. No differences were observed for IFN-α (91.1±3.6 versus 109.1±7.6 pg/ml for respectively WT and ChemR23^−/−^ pDCs; p>0.05), and IL-12 p40 (2256±23 versus 2303±27 pg/ml for respectively WT and ChemR23^−/−^ pDCs; p>0.05). Data represent the mean +/− SEM (n = 3).
(TIF)

Figure S6 **Chemerin has no chemotactic activity for pDCs purified from ChemR23-deficient mice.** (**A**) PDCs were purified from spleen cells as described. Over 90% of the purified cells were identified as CD11b^−^ CD11c^+^ Gr-1^+^ mPDCA^+^ cells, which correspond to pDCs. (**B**) A chemotaxis assay was performed with pDCs from wild-type (WT) (left panel) and ChemR23^−/−^ mice (right panel) using a 48-well microchemotaxis Boyden chamber with polycarbonate membranes (5 µm pores). The cell suspension (10^4^ cells in 50 µl) was placed in the upper chamber. The lower wells contained 30 µl of medium (RPMI) with different concentrations of mouse recombinant chemerin or CXCL12 (used as positive control), and the chamber was incubated at 37°C for 90 min. The membrane was removed and cells that migrated to the lower side of the membrane were washed, fixed, stained with Hoechst, and counted with the ImageJ software. All conditions were tested in triplicate. Controls were performed in the absence of chemoattractant in the lower wells. The results were expressed as migration ratio (mean cell number per well with chemoattractant over mean cell number per well in the absence of chemoattractant). As expected, only pDCs purified from WT mice migrated with a classical bell-shaped curve in response to increasing amounts of recombinant chemerin, with a peak corresponding to 0.1 and 1 nM concentrations.
(TIF)

Figure S7 **IL-6 and IFN-γ levels in BAL fluid of ChemR23^−/−^ mice receiving pDCs from either wild-type or ChemR23^−/−^ mice.** 10^6^ pDCs from wild-type (WT) or ChemR23^−/−^ (KO) mice were transferred intravenously into ChemR23^−/−^ mice at the time of infection (n = 7 per group). Infected WT and KO mice receiving a saline solution were used as controls (n = 5 per group). Mice were sacrificed 14 days after infection and cytokine levels were analyzed in BAL fluids using ELISA. Histograms show the mean ± SEM of IL-6 and IFN-γ levels expressed as pg/ml. *, p<0.05; **, p<0.01, ***, p<0.001.
(TIF)

Table S1 **Sequence of primers used for quantitative RT-PCR.**
(DOC)
