# Supplementary figures and images for: ChemR23 Dampens Lung Inflammation and Enhances Anti-viral Immunity in a Mouse Model of Acute Viral Pneumonia
Source: PLoS Pathog. 2011 Nov 3;7(11):e1002358. doi: 10.1371/journal.ppat.1002358 (PMC3207933; doi:10.1371/journal.ppat.1002358)

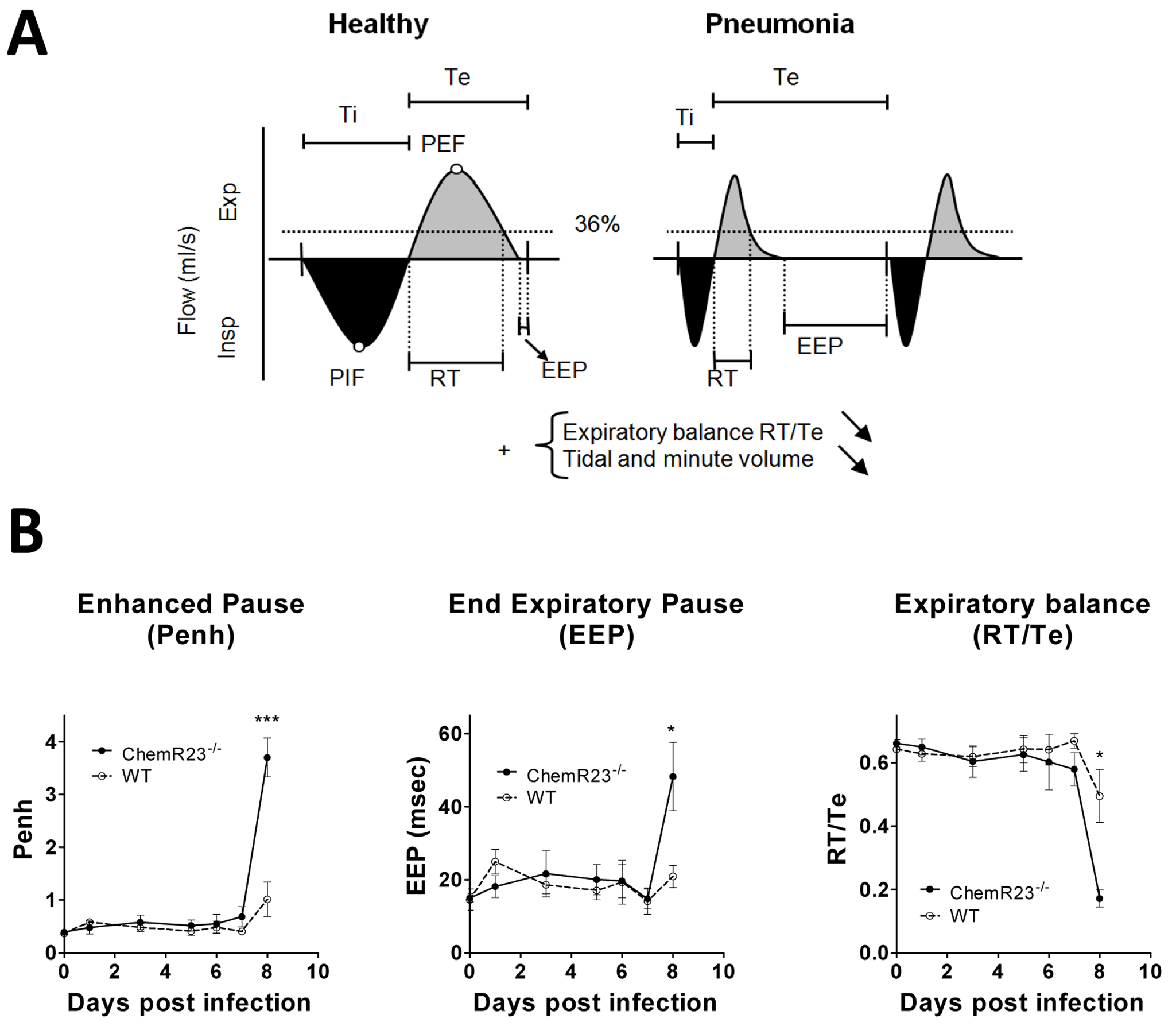

Supplement: Figure S1 — Assessment of respiratory functional parameters in PVM-infected wild-type and ChemR23−/− mice using a whole-body double chamber plethysmograph. Lung function was assessed using a double chamber plethysmograph. (A) Awake mice were placed between the two compartments (nasal and thoraco-abdominal) of the instrument, flow variations were recorded and the following functional parameters were determined: inspiratory time (Ti); expiratory time (Te); peak inspiratory flow (PIF); peak expiratory flow (PEF); tidal volume (TV); expiratory balance (RT/Te), in which RT is the relaxation time determined as the time needed to expire 64% of the inspired volume; end expiratory pause (EEP), Te - RT; and enhanced pause (Penh), ((TE/RT)-1)x(PEF/PIF) (E4). (B) Before and at selected time points post-infection, ChemR23−/− (closed circles) and wild-type (WT) (open circles) mice were assessed for respiratory function. Whereas no significant changes were observed in WT mice, ChemR23−/− mice displayed severe changes at days 7 and 8 post-infection, consistent with marked respiratory dysfunction and more specifically a restrictive syndrome. Along with a reduction in tidal volume, ChemR23−/− mice displayed a 2.3-fold increase in end expiratory pause (p<0.05), a 3.4-fold increase in enhanced pause (Penh) (p<0.001), and a 65% reduction in expiratory balance (p<0.05). The displayed data are the mean ± SEM for groups of at least four animals, and are representative of two independent experiments. *, p<0.05; ***, p<0.001. (TIF) [file ppat.1002358.s001.tif]

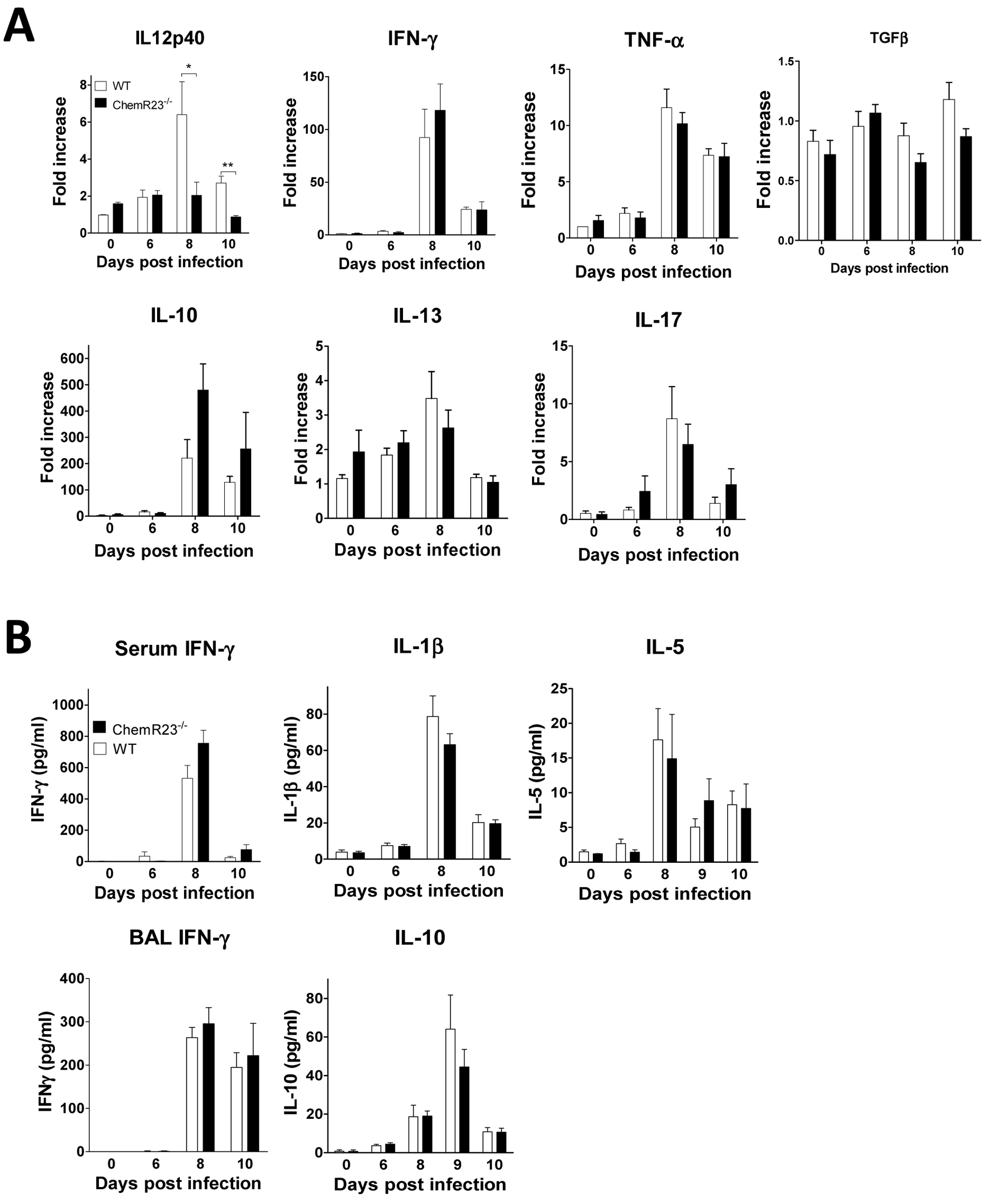

Supplement: Figure S2 — Cytokinic profile in lung determined by ELISA and quantitative RT-PCR. (A) Lung total RNA was extracted, purified and reverse transcribed into cDNA that was analyzed by quantitative real-time PCR. Sequences of primer pairs are displayed in Table S1. The data were normalized using two housekeeping genes (YWHAZ and CANX) as references, and reported to the corresponding transcript level in uninfected control mice. Assayed cytokines were TNF-α, IL-12p40, IL-10, IL-13, IL-17, TGF-β and IFN-γ. When gene expression was upregulated during the course of infection, a peak value was obtained at day 8 post-infection without significant differences between wild-type (WT) and ChemR23−/− mice, except for IL-12p40 (∼3-fold higher values at days 8 and 10 post-infection in ChemR23−/− mice; p<0.05). (B) IFN-γ in serum and BAL fluids, as well as IL-1β, IL-5 and IL-10 in lung homogenates were measured by ELISA in ChemR23−/− and WT mice before and at various time points after PVM infection. No significant difference was observed for these cytokines. Data are the mean ± SEM for groups of at least five animals. *, p<0.05; **, p<0.01. (TIF) [file ppat.1002358.s002.tif]

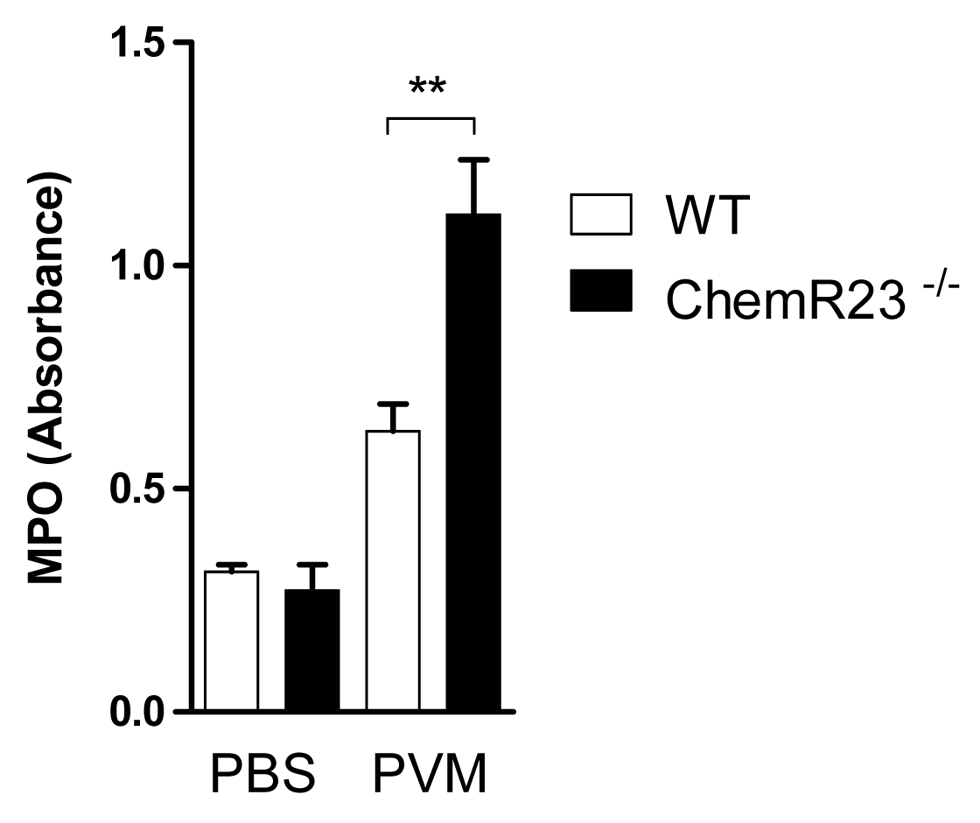

Supplement: Figure S3 — Higher myeloperoxydase activity in the lung of infected ChemR23-deficient mice. Myeloperoxydase (MPO) activity was assayed in lung homogenates from wild-type (WT) and ChemR23−/− mice, 9 days after viral inoculation or PBS instillation. Cell pellets from homogenized lungs were resuspended in PBS containing 13.7 mM of hexadecyltrimethyl ammonium bromide (HTAB) and 5 mM EDTA. Following centrifugation, supernatants were harvested and diluted in Hanks' balanced salt solution (HBSS) containing 1 mM HTAB, 0.4 mM EDTA, 0.15 mM of o-dianisidine dihydrochloride solution and 0.56 mM of H2O2. After 15 minutes at 37°C, the reaction was stopped with 25 µl of 1% NaN3. The MPO activity was determined by measuring the absorbance at 460 nm against medium. No difference was observed between uninfected WT and ChemR23−/− mice, whereas infected ChemR23−/− mice presented a significantly higher MPO activity than infected WT mice. The data represent the mean ± SEM for groups of seven animals and are representative of three independent experiments. **, p<0.01. (TIF) [file ppat.1002358.s003.tif]

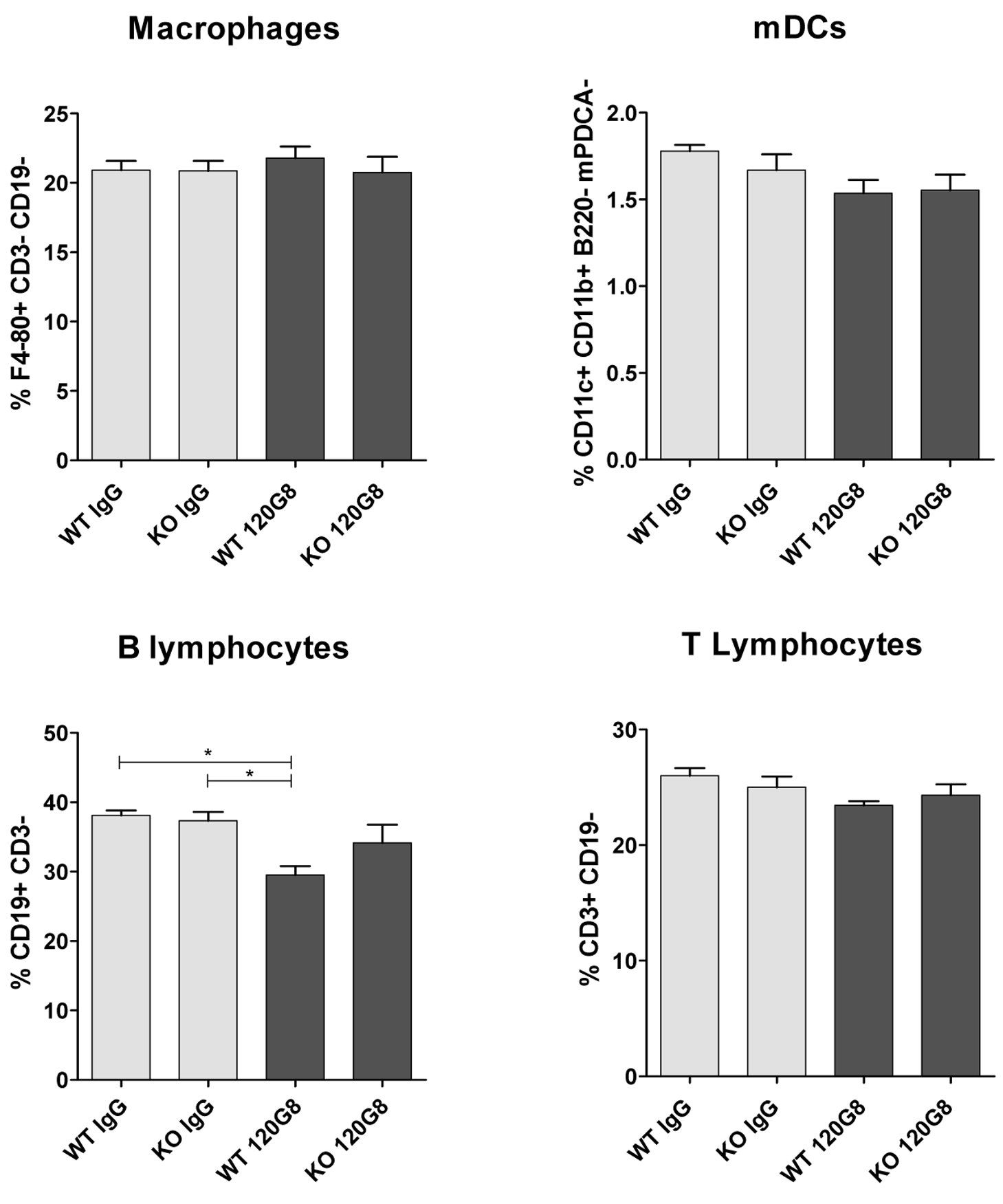

Supplement: Figure S4 — Assessment of the specificity of pDC depletion by the 120G8 antibody. To investigate the role of pDCs in the stronger inflammatory response observed in infected ChemR23−/− mice, pDC depletion experiments were performed. This was achieved by using a commercially available antibody (120G8). To assess the specificity of this depletion procedure, spleens from wild-type (WT) and ChemR23−/− (KO) mice treated or not by the 120G8 antibody were obtained at day 8 post-infection. Cells were isolated and stained for myeloid dendritic cells (mDCs) (CD11c+ CD11b+ B220− mPDCA−), macrophages (F4-80+ CD11b+ CD11c−), T (CD3+ CD19− F4-80−) and B (CD19+ CD3− F4-80−) lymphocytes. There was a mild but significant (p<0.05) decrease in the proportion of B cells in depleted WT (but not ChemR23−/−) mice, as compared to control IgG-treated mice. No effect of the depleting antibody was observed for mDCs, macrophages and T cells. Data are the mean ± SEM for groups of five animals. * = p<0.05. (TIF) [file ppat.1002358.s004.tif]

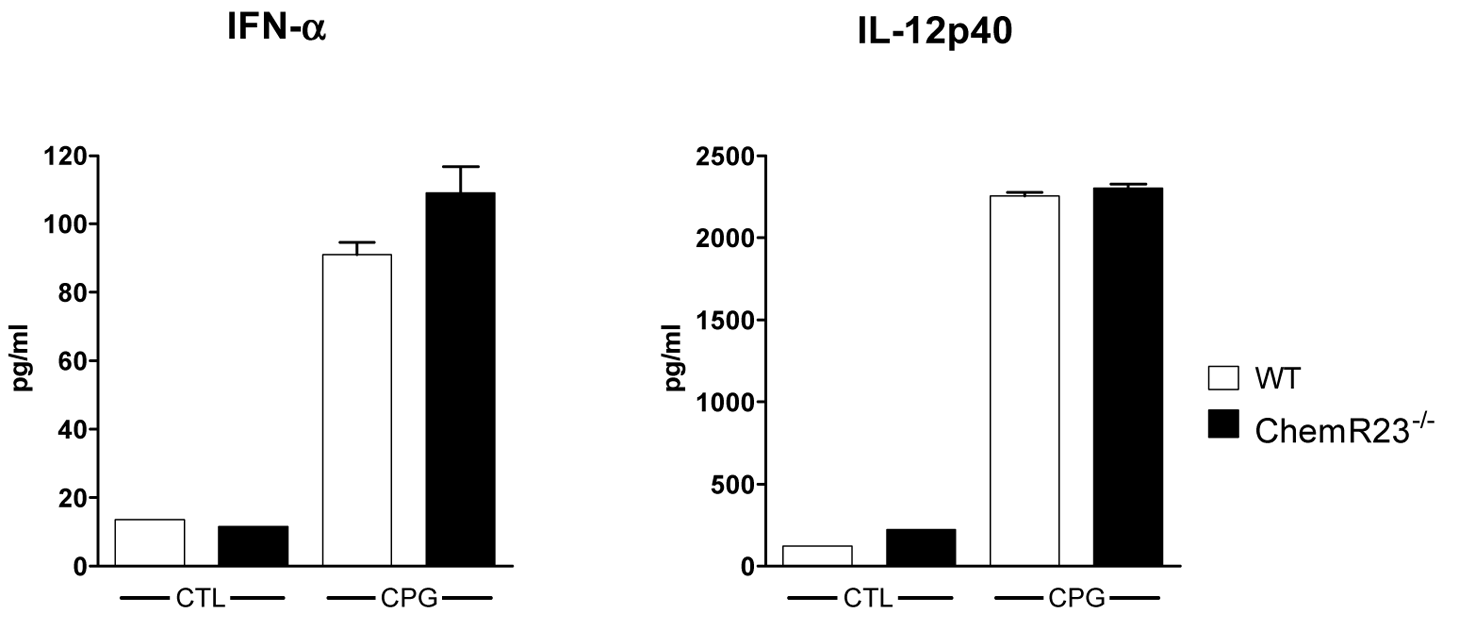

Supplement: Figure S5 — Production of IFN-α and IL-12p40 is not impaired in ChemR23−/− pDCs. The capacity of pDCs purified from wild-type (WT) and ChemR23−/− mice to synthesize and secrete type I IFNs and IL-12p40 subunit was studied following stimulation by CpG (a TLR9 agonist). pDCs were prepared from spleen cells as described, resuspended at a density of 105 cells/ml, stimulated in vitro by CpG (3 µg/ml), and the production of IFN-α and IL-12p40 was assayed by ELISA on the supernatant collected 24 hours later. Experiments were performed in triplicates. No differences were observed for IFN-α (91.1±3.6 versus 109.1±7.6 pg/ml for respectively WT and ChemR23−/− pDCs; p>0.05), and IL-12 p40 (2256±23 versus 2303±27 pg/ml for respectively WT and ChemR23−/− pDCs; p>0.05). Data represent the mean +/− SEM (n = 3). (TIF) [file ppat.1002358.s005.tif]

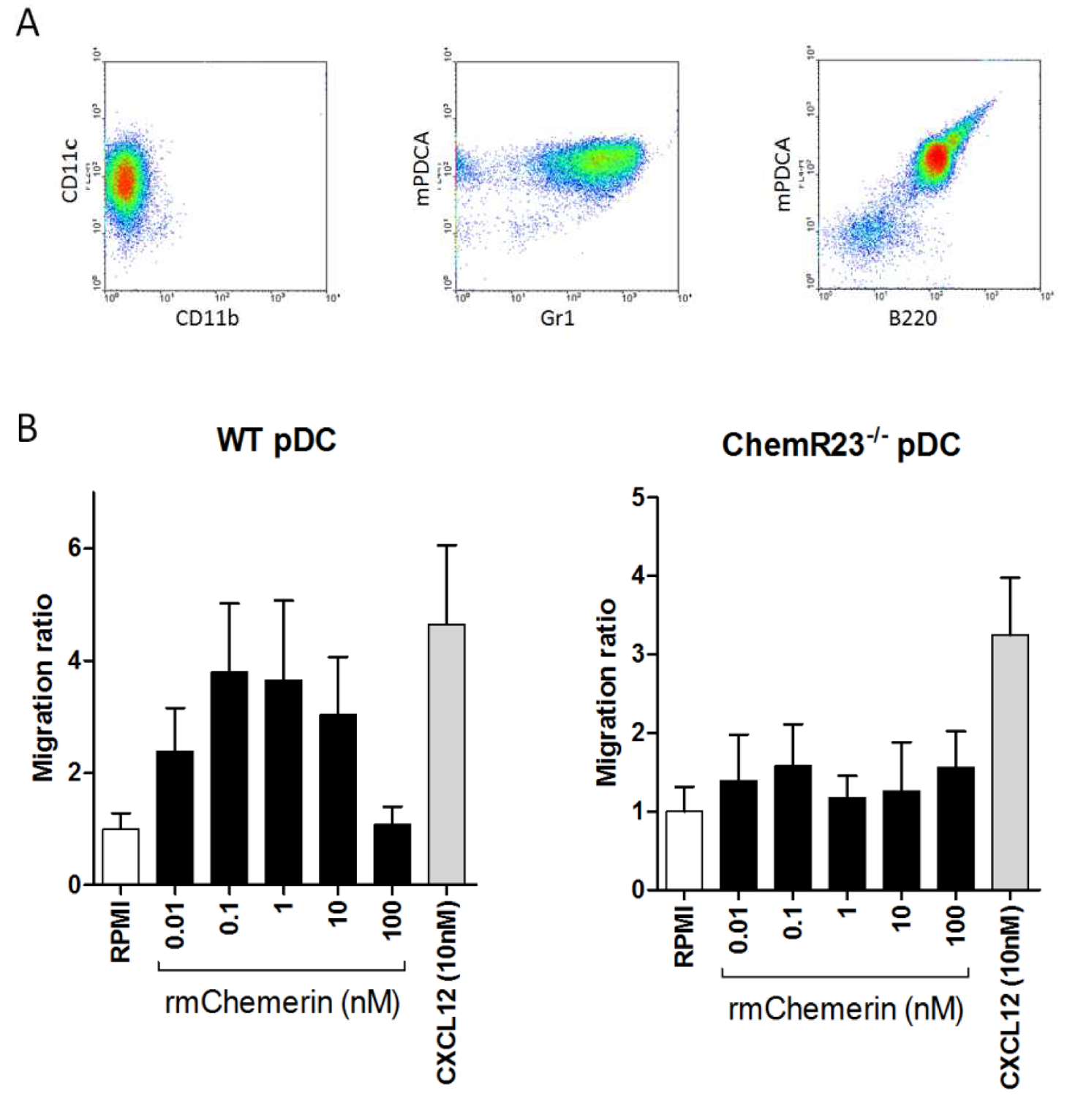

Supplement: Figure S6 — Chemerin has no chemotactic activity for pDCs purified from ChemR23-deficient mice. (A) PDCs were purified from spleen cells as described. Over 90% of the purified cells were identified as CD11b− CD11c+ Gr-1+ mPDCA+ cells, which correspond to pDCs. (B) A chemotaxis assay was performed with pDCs from wild-type (WT) (left panel) and ChemR23−/− mice (right panel) using a 48-well microchemotaxis Boyden chamber with polycarbonate membranes (5 µm pores). The cell suspension (104 cells in 50 µl) was placed in the upper chamber. The lower wells contained 30 µl of medium (RPMI) with different concentrations of mouse recombinant chemerin or CXCL12 (used as positive control), and the chamber was incubated at 37°C for 90 min. The membrane was removed and cells that migrated to the lower side of the membrane were washed, fixed, stained with Hoechst, and counted with the ImageJ software. All conditions were tested in triplicate. Controls were performed in the absence of chemoattractant in the lower wells. The results were expressed as migration ratio (mean cell number per well with chemoattractant over mean cell number per well in the absence of chemoattractant). As expected, only pDCs purified from WT mice migrated with a classical bell-shaped curve in response to increasing amounts of recombinant chemerin, with a peak corresponding to 0.1 and 1 nM concentrations. (TIF) [file ppat.1002358.s006.tif]

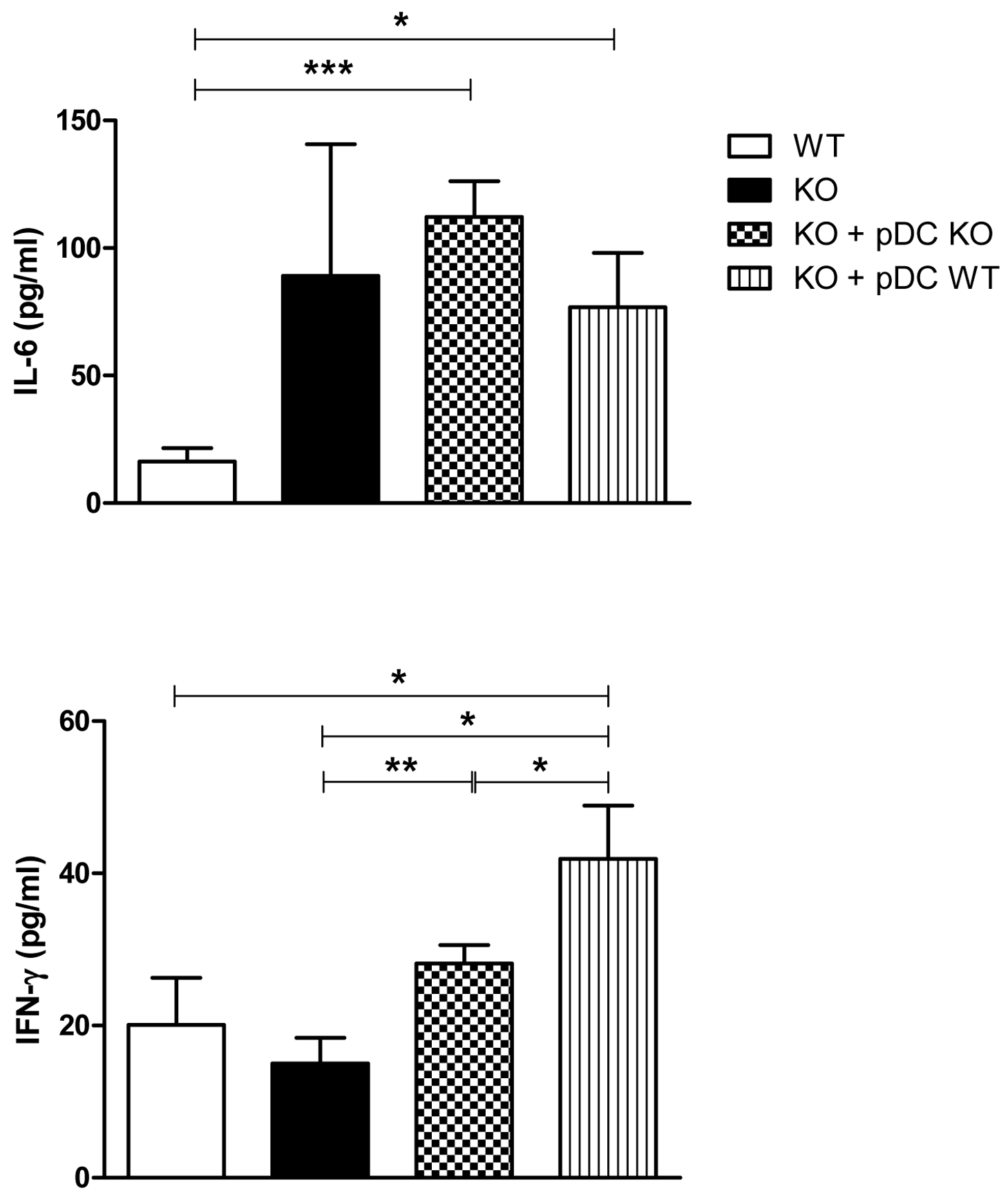

Supplement: Figure S7 — IL-6 and IFN-γ levels in BAL fluid of ChemR23−/− mice receiving pDCs from either wild-type or ChemR23−/− mice. 106 pDCs from wild-type (WT) or ChemR23−/− (KO) mice were transferred intravenously into ChemR23−/− mice at the time of infection (n = 7 per group). Infected WT and KO mice receiving a saline solution were used as controls (n = 5 per group). Mice were sacrificed 14 days after infection and cytokine levels were analyzed in BAL fluids using ELISA. Histograms show the mean ± SEM of IL-6 and IFN-γ levels expressed as pg/ml. *, p<0.05; **, p<0.01, ***, p<0.001. (TIF) [file ppat.1002358.s007.tif]
