# Supplementary material for: ChemR23 Dampens Lung Inflammation and Enhances Anti-viral Immunity in a Mouse Model of Acute Viral Pneumonia
Source: PLoS Pathog. 2011 Nov 3;7(11):e1002358. doi: 10.1371/journal.ppat.1002358 (PMC3207933; doi:10.1371/journal.ppat.1002358)
Supplement: Table S1 — Sequence of primers used for quantitative RT-PCR. (DOC) [file ppat.1002358.s008.doc]

**Table S1. Sequence of primers used for quantitative RT-PCR.**

| Genes |  | Primer sequences |
| --- | --- | --- |
| Chemerin | Forward  Reverse | 5’-CCAACTGCCCCAAGAAGGA-3’  5’-AATGCAGGCCAGGCATTTC-3’ |
| IFN-α | Forward  Reverse | 5’-GACTTTGGATTTCCCCTGGAG-3’  5’-AAGCCTTTGATGTGAAGAGGTTC-3’ |
| IFN-β | Forward  Reverse | 5’-GTTACACTGCCTTTGCCATCC-3’  5’-CAACAATAGTCTCATTCCACCCAG-3’ |
| IFN-γ | Forward  Reverse | 5’-GGCTGTCCCTGAAAGAAAGC-3’  5’-AGCGAGTTATTTGTCATTCGG-3’ |
| IL-12p40 | Forward  Reverse | 5’-GAGCACTCCCCATTCCTACT-3’  5’-CCCTCCTCTGTCTCCTTCAT-3’ |
| IL-17 | Forward  Reverse | 5’-CTCAGACTACCTCAACCGTTC-3’  5’-TTCAGGACCAGGATCCTTGC-3’ |
| TNF-α | Forward  Reverse | 5’-ACCCTCACACTCAGATCATC-3’  5’-GAGTAGACAAGGTACAACCC-3’ |
| IL-10 | Forward  Reverse | 5’-GCCACATGCTCCTAGAGCTG-3’  5’-CAGCTGGTCCTTTGTTTGAAA-3’ |
| IL-13 | Forward  Reverse | 5’-CCTGGCTCTTGCTTGCCTT-3’  5’-GGTCTTGTGTGATGTTGCTCA-3’ |
| TGF-β1 | Forward  Reverse | 5’-CCGAAGCGGACTACTATGCTA-3’  5’-TTTCTCATAGATGGCGTTGTTG-3’ |
| CANX | Forward  Reverse | 5’-TTGCTGACTCCTTTGACAGAGG-3’  5’-CCACTTTCCATCATATTTGGCA-3’ |
| YWHAZ | Forward  Reverse | 5’-TGCAACGATGTACTGTCTCTTTTG-3’  5’-CGGTAGTAGTCACCCTTCATTTTCA-3’ |
